# Uncovering bacterial-mammalian cell interactions via single-cell tracking

**DOI:** 10.1186/s12915-024-02056-z

**Published:** 2024-11-11

**Authors:** Narendra K. Dewangan, Sayed Golam Mohiuddin, Shayne Sensenbach, Prashant Karki, Mehmet A. Orman

**Affiliations:** William A. Brookshire Department of Chemical and Biomolecular Engineering, University of Houston, Houston, TX 77204-4004 USA

**Keywords:** Host–pathogen interactions, Bacterial adhesion, Bacterial motility, Single-cell tracking, Lung cells, Skin cells, Antibiotic tolerance

## Abstract

**Background:**

The interactions between bacterial pathogens and host cells are characterized by a multitude of complexities, leading to a wide range of heterogeneous outcomes. Despite extensive research, we still have a limited understanding of how bacterial motility in complex environments impacts their ability to tolerate antibiotics and adhere to mammalian cell surfaces. The challenge lies in unraveling the complexity of these interactions and developing quantitative microscopy approaches to predict the behavior of bacterial populations.

**Results:**

To address this challenge, we directed our efforts towards *Pseudomonas aeruginosa*, a pathogenic bacterium known for producing thick films in the lungs of cystic fibrosis patients, and *Escherichia coli*, used as a proof of concept to develop and demonstrate our single-cell tracking approaches. Our results revealed that *P. aeruginosa* exhibits diverse and complex interactions on mammalian cell surfaces, such as adhesion, rotational motion, and swimming, unlike the less interactive behavior of *Escherichia coli*. Our analysis indicated that *P. aeruginosa* demonstrated lower mean-squared displacement (MSD) values and greater adherence to mammalian cells compared to *E. coli*, which showed higher MSD slopes and less frequent adherence. Genetic mutations in membrane proteins of *P. aeruginosa* resulted in altered displacement patterns and reduced adhesion, with the Δ*fliD* mutant displaying a more Gaussian displacement distribution and significantly less adherence to mammalian cells. Adhesion and tolerance mechanisms are diverse and complex, potentially involving distinct pathways; however, our findings highlight the therapeutic potential of targeting the *fliD* gene (encoding a critical flagellum protein), as its deletion not only reduced adherence but also antibiotic tolerance.

**Conclusions:**

Overall, our findings underscore the importance of single cell tracking in accurately assessing bacterial behavior over short time periods and highlight its significant potential in guiding effective intervention strategies.

**Supplementary Information:**

The online version contains supplementary material available at 10.1186/s12915-024-02056-z.

## Background

Antibiotics are commonly used to control bacterial infections in host organisms. However, treating infections caused by pathogenic bacteria like *Pseudomonas aeruginosa* can be difficult due to their efficient adhesion to human tissues [1, 2]. This poses a significant risk to patients with cystic fibrosis (CF), as this bacterium can produce thick biofilms in their lungs. Bacterial attachment to epithelial cells is crucial and represents the first step in biofilm formation. In CF patients, mutations in the cystic fibrosis transmembrane conductance regulator (CFTR) result in increased materials that promote bacterial adhesion to epithelial cells [3]. This highlights the critical role of cell adhesion on the lung surfaces in CF patients [3]. To combat these pathogens, alternative strategies are being explored, including studying their adhesion to host surfaces and the subsequent biofilm formation. Biofilms, which are composed of bacterial colonies surrounded by an extracellular polymer substance, provide a protective shield against antimicrobial agents, making bacteria within them more tolerant to antibiotics than their free-living (i.e., planktonic) counterparts [2]. Bacterial motility, chemotaxis, and adhesion molecules play a vital role in this process [4–6], and bacteria can be guided to surfaces through intricate and varied mechanisms [4].

*P. aeruginosa* might be more adhesive to biotic or abiotic surfaces than other microbial cells, including *Escherichia coli*, due to its specialized adhesion molecules (fimbriae, pili, flagella), robust biofilm formation capabilities, exopolysaccharide production, and specific interactions with host cell receptors [7–9]. The adhesion of gram-negative bacteria, such as *E. coli*, to host surfaces is significantly enhanced by the presence of fimbriae. These structures, which are shorter and thinner than pili, enable specific binding of proteins like FimH to receptors on mammalian cells, thereby facilitating bacterial attachment and colonization [10–12]. This process is made possible by other crucial fimbrial proteins, including FimT and FimU, which are involved in fimbrial biogenesis [13]. The chaperone-usher systems (CupA, CupB, CupC, CupD, and CupE) and type IV pili, consisting of flexible filaments with a rod-like structure measuring 1–2 μm in length and 5–8 nm in diameter [14], are crucial in facilitating *P. aeruginosa* adhesion to surfaces and promoting the formation of antibiotic-tolerant persistent biofilms [15, 16]. *P. aeruginosa* assembles both type IVa and type IVb pili, which are differentiated by the variations in their major and minor pilin subunits that make up the pilus fiber [17]. The *pilA* pilin protein is oligomerized into a helix and is a major binding receptor responsible for pili-mediated adhesion to surfaces [18–20], as the deletion of *pilA* mutant impairs bacterial adhesion abilities [19, 21]. Additionally, type IV pili also play a role in twitching motility [22], and genetic perturbations in pili genes were found to reduce the cytotoxicity of bacteria towards mammalian cells, with the *pilA* mutant exhibiting the lowest cytotoxicity [23].

The interaction of bacteria with surfaces is influenced by various microscopic forces, including van der Waals interactions, electrostatic attraction or repulsion, hydrophobic interactions, and acid–base interactions. The capability of bacteria to move has a positive impact on their adherence to both biotic and abiotic surfaces [5, 24, 25]. Bacterial motility is frequently facilitated by the flagellum, a long, slender filament that acts as a whip-like organelle. This adaptation together with adhesive properties of cell surface components and appendages (i.e., adhesins like fimbria and pili) provide bacteria with a competitive advantage in overcoming the electrostatic repulsion between the cell body and surfaces [13]. To reduce bacterial adhesion and infection, anti-adhesin therapy can be employed, in which small molecules bind to bacterial adhesins [26, 27]. However, this approach can be challenging as multiple anti-adhesins may be required to significantly reduce adhesion. Anti-adhesin-based antibody vaccines, such as those developed against *P. aeruginosa*, are an emerging strategy to fight bacterial infections.

Although targeting adhesins and motility in bacteria through drug development holds promise as a potential alternative to antibiotics or as a complementary approach to prevent bacterial drug tolerance, our current comprehension of host and pathogen cell interactions, as well as their molecular level heterogeneity, falls short. The mechanisms behind antibiotic-tolerant cells have primarily been explored in free-floating bacteria (planktonic systems) [28], but the influence of bacterium-mammalian cell interactions on bacterial cell adherence and tolerance remains largely unknown. The objective of this project is to gain a deeper understanding of these interactions by developing a quantitative microscopy approach. This approach facilitated the comparison of adhesion and motility characteristics between different organisms (*E. coli* vs *P. aeruginosa*) and different genetic perturbations within *P. aeruginosa*, providing insights into bacterial behavior at the host–pathogen interface.

## Results

### Single-cell tracking reveals distinct behavioral dynamics and population-level characteristics of bacterial cells on mammalian cell surfaces

*P. aeruginosa* is known for its adherence to biotic and abiotic surfaces [7], whereas *E. coli*, used here as a model to develop our methods, is less prone to surface attachment [8]. Our preliminary investigations, which utilized fluorescence microscopy and single-cell tracking techniques to analyze interactions between a free-floating bacterium and a single lung cell (H1975), revealed highly heterogeneous interactions between *P. aeruginosa* and mammalian cells, compared to *E. coli*. We observed that some *P. aeruginosa* cells stop moving after adhering to the mammalian cell surface; others rotate while tethered, swim while partially adhered, or swim towards the mammalian cell before abruptly turning away (Additional file 1: Fig. S1a-d and Additional file 2: Movie S1a-d). Some adhered bacteria do not significantly alter their orientation and have a slope of the mean-squared displacement (MSD) curve much less than 1 (Additional file 1: Fig. S1a, c, e, g, i, k) while swimming bacteria can exhibit changes in orientation angle and a slope of the MSD curve around or greater than 1 (Additional file 1: Fig. S1d, h, l). A tethered cell may have a rotational motion that may result in an oscillatory angular behavior between 90° and − 90°, while MSD remains less than 1 (Additional file 1: Fig. S1b, f, j and Additional file 2: Movie S1b). Despite the difficulty in studying the impact of these diverse and redundant interactions on antibiotic tolerance, we opted to monitor bacterial cell trajectories at the single-cell resolution on a surface that is completely covered with mammalian cells and categorize the trajectories based on the MSD data. We chose to focus on MSD data because other interaction properties, such as the angular displacement behaviors of bacteria, are highly complex and hard to categorize in a 2-dimensional system (Additional file 1: Fig. S1).

**Fig. 1:**
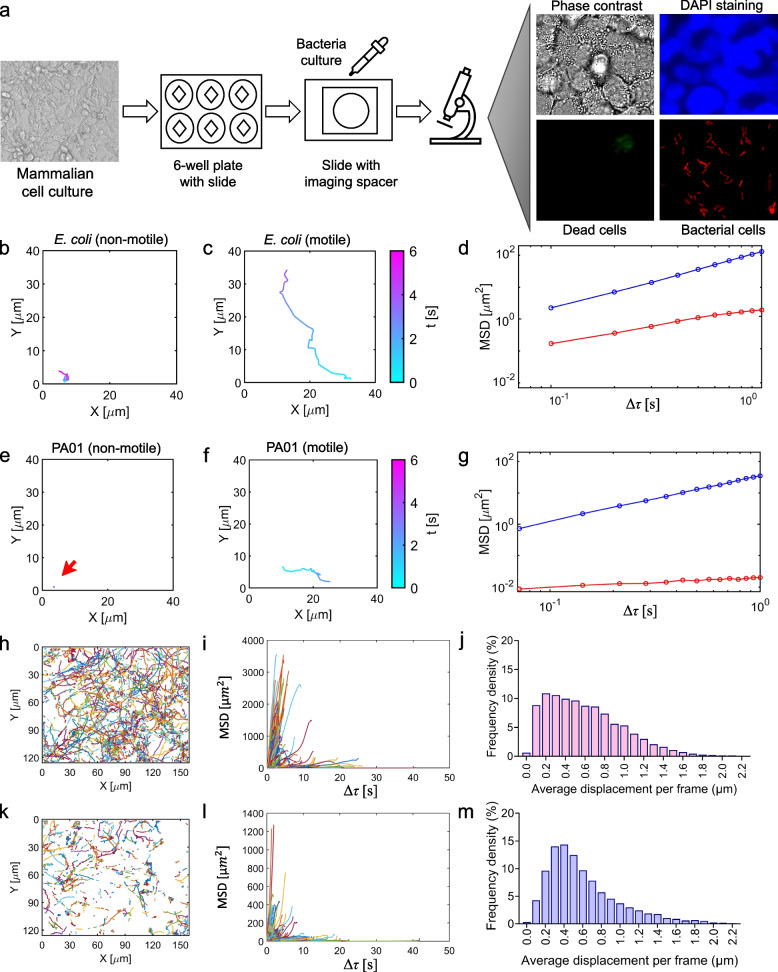
Bacterial cell trajectories. **a** A representative image was presented to illustrate the experimental approach used for monitoring the interactions between mammalian and bacterial cells. Nuclei and dead mammalian cells were stained using DAPI and green fluorescent dyes, while bacterial cells were detected using the mCherry expression system (red fluorescent). **b** The trajectory of an *E. coli* MG1655 cell that exhibits lower displacement compared to swimming cells. **c** The trajectory of a swimming *E. coli* cell. **d** The mean square displacements (MSDs) for the *E. coli* cell trajectories shown in panels **b** (red) and **c** (blue) on a logarithmic scale. **e** The trajectory of a *P. aeruginosa* PAO1 cell that experiences lower displacement than swimming cells. The red arrow highlights the trajectory of the cell. **f** The trajectory of a swimming *P. aeruginosa* cell. **g** The MSDs for the *P. aeruginosa* cell trajectories shown in panels **e** (red) and **f** (blue) on a logarithmic scale. **h**–**j** The trajectory analysis of *E. coli* cells at the population level, revealing the relationship between individual trajectories (**h**), the MSD of each individual cell (**i**), and the distribution of cell displacements (**j**). Displacement distribution histograms were constructed by binning average displacements into equal-width bins, counting cell frequencies per bin, and normalizing counts for probability distribution (see the “Methods” section). **k**–**m** The trajectory analysis of *P. aeruginosa* cells at the population level (**k** cell trajectories; **l** the MSD of each individual cell; **m** the displacement distribution histogram). The data presented in these panels correspond to a minimum of three biological replicates, with multiple movies captured in each replicate to ensure the analysis of 12,986 *E. coli* and 9219 *P. aeruginosa* cell trajectories

For these experiments, a lung cell-coated coverslip was prepared by incubating the coverslip with mammalian cells with Modified Eagle Medium F12 (DMEM/F12) for 2 days (Fig. 1a). After removing unattached cells by washing the coverslip with phosphate-buffered saline (PBS), a droplet of bacterial suspension was added to a glass slide and covered with the lung cell-coated coverslip using vacuum grease. Movies of bacteria near the surface were captured at a minimum of 22 frames per second (fps). Bacterial cells with an inducible *mCherry* expression cassette were monitored using red fluorescence, while blue 4′,6-diamidino-2-phenylindole (DAPI) nuclear staining and green live/dead staining as well as phase contrast imaging were used to check mammalian cell integrity and to confirm complete coverage of the coverslip with mammalian cells (Fig. 1a). This complete coverage is crucial for preventing bacteria from becoming trapped within the interstitial spaces among mammalian cells and to hinder direct adherence of bacterial cells to the coverslip. Also, mammalian cells within their natural host environment are arranged in layers, rendering this configuration highly physiologically relevant. The representative trajectories of swimming and adhered bacteria (both *Escherichia coli* and *P. aeruginosa*) on the lung cell line are shown in Fig. 1b–g. The bacterial trajectories showed distinct patterns, including long trajectories corresponding to swimming bacteria (Fig. 1c, f), and shorter trajectories representing slow-moving or attached bacteria (Fig. 1b, e). Our observations revealed that cells that potentially adhere to a lung cell surface exhibit an average displacement of less than 1 pixel per frame (1 pixel = 0.12 μm), whereas cells that swim exhibit significantly greater displacements. The slower-moving *P. aeruginosa* bacterium is likely adhered to the lung cell surface, resulting in a significantly lower slope of its MSD as compared to that of *E. coli* (Fig. 1d, g). Additionally, among swimming bacteria, *E. coli* exhibits a longer trajectory with larger displacements and a higher MSD slope than those of *P. aeruginosa* cells (Fig. 1d, g), possibly due to less attractive forces experienced by *E. coli* towards the mammalian-cell surface, compared to those experienced by *P. aeruginosa*.

To characterize single-cell behaviors at the population level, we tracked thousands of *P. aeruginosa* and *E. coli* cells during their interaction with mammalian cells (Fig. 1h, k, and Additional file 2: Movies S2a-d) and observed that the MSD values of individual *P. aeruginosa* cells are generally lower than those of *E. coli* cells (Fig. 1i, l). Due to the diverse behaviors exhibited by cells in response to their environment, resulting in a wide range of displacement values (Fig. 1i, l), we generated population-level cell displacement histograms (see the “Methods” section for details) using the single-cell motions of *P. aeruginosa* and *E. coli*. While we did not observe multimodal distributions in displacement data, both *P. aeruginosa* and *E. coli* data exhibited non-normal or non-Gaussian distributions with noticeable skewness (Fig. 1j, m), which may be expected, as cell movement is often influenced by non-linear processes associated with cell adhesive properties, directional motions, or stochastic fluctuations in movement [29–31]. As the majority of *P. aeruginosa* cells experience smaller displacements with fewer cells showing larger displacements, compared to the *E. coli* population where more cells exhibit larger displacements (Fig. 1j, m), the empirical cumulative distribution functions between the two cell populations were found to be significantly different (*P* < 0.000001). We employed the Kolmogorov–Smirnov test for this analysis, a non-parametric method that does not assume a specific distribution shape, making it suitable for comparing distributions commonly observed in biological data [32, 33]. *P. aeruginosa* trajectories may show directional movement towards surfaces, suggesting they experience more constrained motion due to interactions with the surface. In contrast, *E. coli* cells, propelled by flagella-driven swimming that allows rapid movement in liquid environments, may exhibit lower adherence frequency to surfaces, and this hypothesis will be tested in the subsequent sections.

### *P. aeruginosa* forms dense layers on the mammalian cell surface

**Fig. 2:**
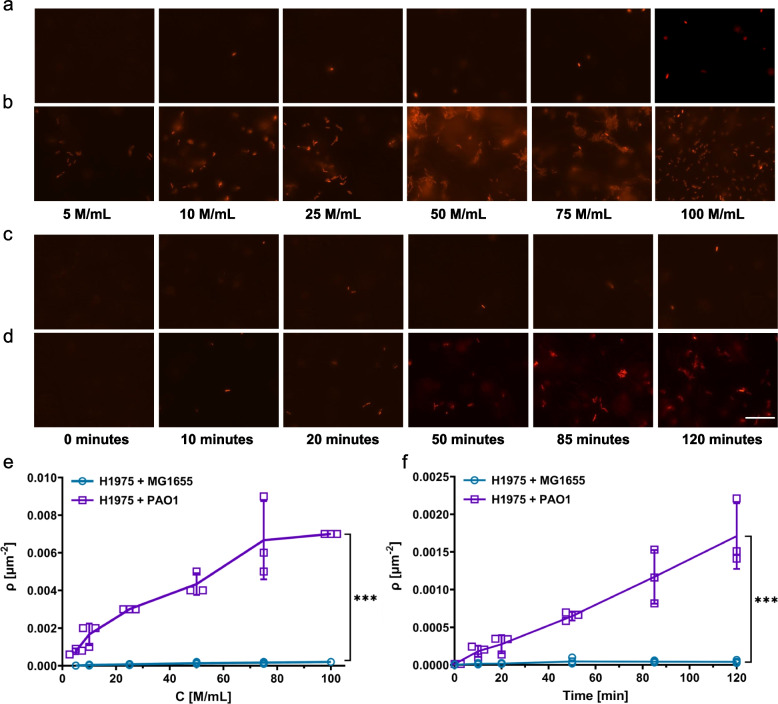
The changes in the density of bacteria on mammalian cell surfaces in relation to the initial concentration of bacterial cells and co-culturing time. **a**, **c** Micrographs of *E. coli* MG16555 on lung cells (H1975) as a function of the number of bacterial cells (M = million) or co-culturing time, respectively. **b**, **d** Micrographs of *P. aeruginosa* PAO1 on lung cells as a function of the number of bacterial cells or co-culturing time, respectively. The scale bar is 30 μm. **e**, **f** The surface density (*ρ*) of bacteria as a function of the number of bacterial cells or co-culturing time, respectively. The surface density is defined as the number of adhered cells per unit surface area. *C* = initial concentration of bacterial cells. The *F*-statistics were used to perform a statistical comparison between two groups, where ***P < 0.001. The data for each time point is represented as the mean value ± standard deviation. *N* = 3

In Fig. 1, we tracked bacterial cells immediately after mixing with mammalian cells, revealing the potential of single cell tracking to predict bacterial adherence behavior during initial interactions with mammalian cells. We believe that short-term interactions can dictate long-term outcomes; therefore, we exposed bacteria to mammalian cells for longer periods to measure surface density (*ρ*), defined as the number of adhered bacteria per unit mammalian-cell surface area. Adhesive bacteria should exhibit higher surface density, which will further validate the expected correlation between bacterial adherence and displacement properties. To test this, we conducted an experiment to investigate how the surface density changes over time or with varying initial bacteria concentrations. First, we exposed lung cells to different initial concentrations of either *E. coli* or *P. aeruginosa* for a period of 2 h. Following the incubation period, we washed the slides three times with PBS and once with DMEM/F12 to completely remove any unattached bacteria before imaging with a fluorescence microscope. Our results show that *P. aeruginosa* adhered on the surface to a greater extent compared to *E. coli* (Fig. 2a *vs.* Figure 2b). As the initial bacterial concentration increased, the surface density of *P. aeruginosa* on the mammalian cells also increased; this trend, however, was not observed for *E. coli* cells (Fig. 2e). To explore the dynamics of bacterial adhesion to mammalian cells, bacterial suspensions were incubated with mammalian cell surfaces for 0, 10, 20, 50, 85, and 120 min (Fig. 2c, d, f). At the start of the incubation period (*t* = 0), neither *E. coli* nor *P. aeruginosa* adhered to the surfaces of lung cells when a suspension of bacteria was applied and then promptly removed within 1 min, without allowing the cells to incubate (Fig. 2c, d, f). The adhesion of *P. aeruginosa* to lung-cell surfaces increased at a much higher rate than that of *E. coli* as incubation time progressed (Fig. 2f). Collectively, these findings validate our argument that *E. coli* cells exhibit lower adherence frequency compared to *P. aeruginosa*, which demonstrates more constrained movement towards surfaces (Fig. 1).

It is unlikely that the adherence of *P. aeruginosa* is a result of bacteria being trapped in the interstitial spaces between mammalian cells, as this would also be observed with *E. coli* cells. However, the level of adherence of *E. coli* cells is significantly less than that of *P. aeruginosa*, and the surface density of *E. coli* remains mostly unchanged over time or with varying initial concentrations (Fig. 2). To check if similar trends were present in other mammalian cell types, we repeated the experiments with A375 skin cells (Additional file 1: Fig. S2). We found that *P. aeruginosa* adhered more on the skin-cell surface compared to *E. coli*, with an increasing surface density as the initial bacterial concentration and incubation time increased (Additional file 1: Fig. S2a-f). However, no such trends were observed for *E. coli* cells (Additional file 1: Fig. S2a-f), consistent with the lung-cell data. It should be noted that the surface densities of *P. aeruginosa* at 75 million (M)/mL and 100 M/mL on skin cells were not depicted in Additional file 1: Fig. S2b as the presence of a high concentration of *P. aeruginosa* led to toxicity and detachment of the mammalian cells from the glass surface. Altogether, these results indicate that *P. aeruginosa* adhesion may not be specific to any particular type of mammalian cells.

### The *fliD* mutant displays the lowest adherence and a more homogeneous cell displacement distribution

**Fig. 3:**
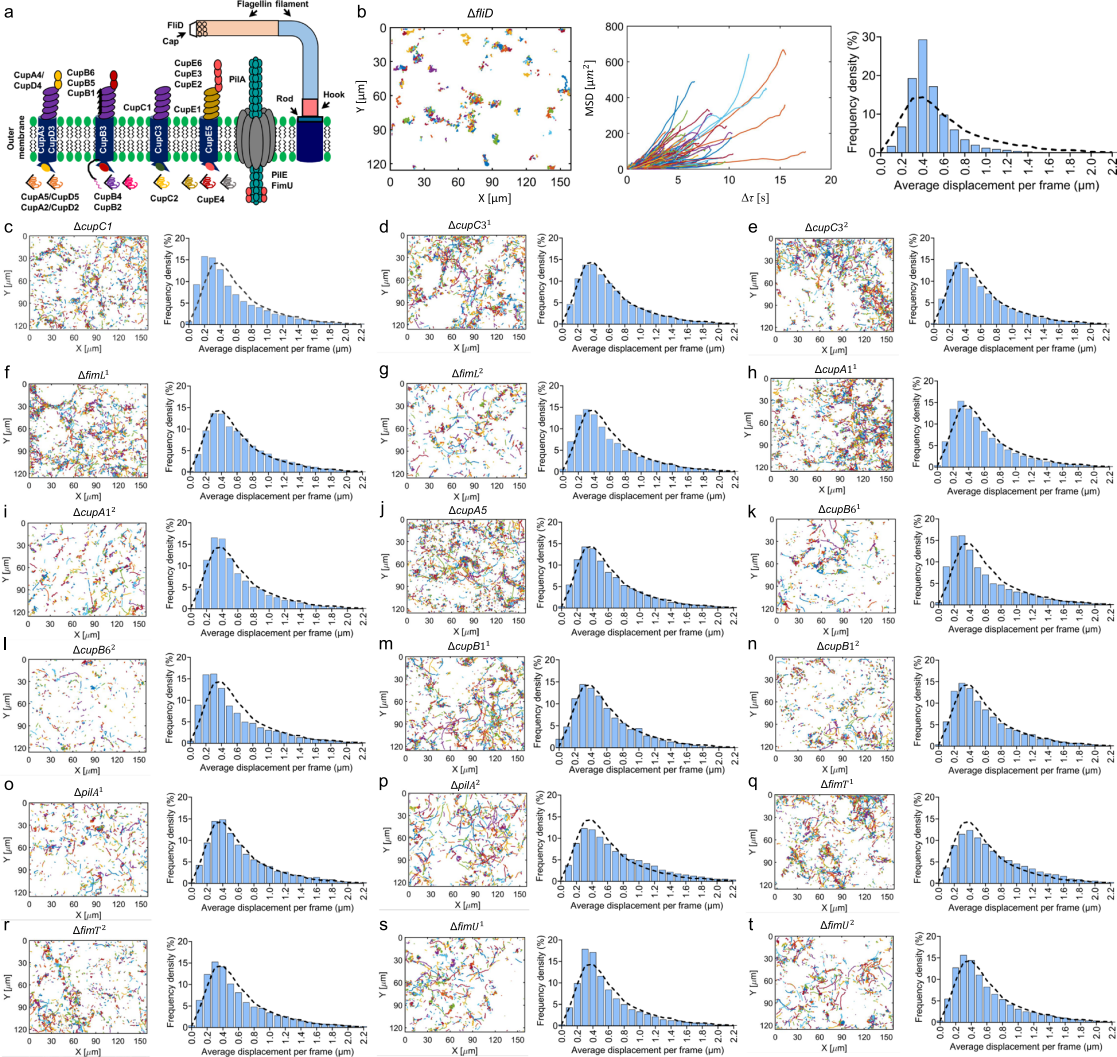
Trajectories and MSDs of *P. aeruginosa* strains on lung-cell surfaces. **a** The panel displays the Chaperone-Usher, pili, and flagellar systems present in *P. aeruginosa* [17, 35–37]. **b** At the population level, cell trajectories, individual MSDs, and the displacement distribution histogram were analyzed for the Δ*fliD* mutant. **c**–**t** At the population level, cell trajectories (see Additional file 1: Fig. S3 for individual MSDs) and displacement distribution histograms were analyzed for the specified mutants of *P. aeruginosa* cells. The dotted lines in the displacement histograms represent the distribution of wild-type *P. aeruginosa* PA01. The data presented in each panel correspond to a minimum of three biological replicates, with multiple movies captured in each replicate, ensuring a sufficient number of cell trajectories were analyzed: Δ*fliD* = 5,600; Δ*cupC1* = 12,553; Δ*cupC3*^*1*^ = 12,539; Δ*cupC3*^*2*^ = 22,005; Δ*fimL*^*1*^ = 15,633; Δ*fimL*^*2*^ = 16,440; Δ*cupA1*^*1*^ = 14,914; Δ*cupA1*^*2*^ = 12,751; Δ*cupA5* = 16,040; Δ*cupB6*^*1*^ = 12,937; Δ*cupB6*^*2*^ = 7375; Δ*cupB1*^*1*^ = 7070; Δ*cupB1*^*2*^ = 13,414; Δ*pilA*^*1*^ = 11,837; Δ*pilA*^*2*^ = 9,837; Δ*fimT*^*1*^ = 13,607; Δ*fimT*^*2*^ = 15,749; Δ*fimU*^*1*^ = 7,862; Δ*fimU*^*2*^ = 11,402 trajectories

Given the observed increased surface density of *P. aeruginosa* on mammalian cells, we directed our efforts towards this pathogenic bacterium, known for forming dense biofilms in the lungs of cystic fibrosis patients. To gain insight into the crucial factors involved in *P. aeruginosa* adhesion to lung cells, we investigated how genetic perturbations on key membrane proteins impact the adherence and displacement properties of *P. aeruginosa* (Fig. 3). The selected strains have mutations in chaperone-usher, pili, and flagellar systems (Fig. 3a), including Δ*cupC1*, Δ*cupC3*^*1*^, Δ*cupC3*^*2*^, Δ*fliD*, Δ*fimL*^*1*^, Δ*fimL*^*2*^, Δ*cupA1*^*1*^, Δ*cupA1*^*2*^, Δ*cupA5*, Δ*cupB6*^*1*^, Δ*cupB6*^*2*^, Δ*cupB1*^*1*^, Δ*cupB1*^*2*^, Δ*pilA*^*1*^, Δ*pilA*^*2*^, Δ*fimT*^*1*^, Δ*fimT*^*2*^, Δ*fimU*^*1*^, and Δ*fimU*^*2*^ (Additional file 1: Table S1). These mutants were obtained from the Manoil Lab and generated using a transposon system [34]. For some strains, there are two distinct mutations (i.e., transposon insertions) in each gene, such as Δ*cupB6*^*1*^ and Δ*cupB6*^*2*^. The genes *cupA1*, *cupB1*, and *cupC1* are responsible for the assembly of the pilus rod [17], with *cupA5* functioning as a chaperone for the transport of pilin and *cupC3* forming the *CupC3* usher assembly and *cupB6* responsible for the expression of the CupB6 receptor protein [17] (Fig. 3a). The gene *pilA* is essential for the assembly of the type of IVa pili and serves as a major functional structure, acting as a grappling hook to enable attachment and movement on surfaces [35] (Fig. 3a). The *fimU*,* fimL*, and *fimT* genes are involved in the formation of minor pilins [36]. The *fliD* gene encodes a crucial cap-shaped protein located at the tip of the bacterial flagellum’s external filament [37]. It plays a key role in organizing the polymerization of flagellin, a filament protein that passes through the center of the flagellum (Fig. 3a).

We monitored the population cell behaviors of the aforementioned strains at the single-cell resolution using our experimental setup and characterized their trajectories based on their MSD values and displacement distributions. Our analysis revealed distinct long and short trajectories in both wild-type and mutant strains indicating the presence of strain-specific motions and interactions (Fig. 3b-t, Additional file 1: Fig. S3). The Δ*fliD* strain displayed trajectories that led to lower single-cell MSD values (Fig. 3b, Additional file 1: Fig. S3). This characteristic of the Δ*fliD* mutant can be ascribed to a lower rate of surface contact and reduced interactions, likely stemming from its reduced motility (Additional file 2: Movie S3). Unlike the other strains, the Δ*fliD* strain may rely on diffusive motion, suggesting that the positions of Δ*fliD* cells may display random variations over time. The majority of strains, including wild-type *P. aeruginosa*, display highly heterogeneous motions, ranging from larger displacements to no displacements (Fig. 1k, l and Fig. 3c-t, Additional file 1: Fig. S3). Furthermore, histogram plots illustrating the average displacement distributions of *P. aeruginosa* mutant strains mostly showed skewness, closely resembling that of the wild type (Fig. 3c-t). However, despite these similarities, most strains exhibited statistically significant differences compared to the wild type (Additional file 1: Table S2), which may be attributed to subtle variations in their distributions. While the skewness similarity suggests some preservation of movement characteristics in mutant strains, the statistical differences may underscore the impact of genetic perturbations on *P. aeruginosa*’s displacement behavior. The most significant change is observed in the Δ*fliD* displacement distribution, exhibiting a behavior closer to Gaussian (Fig. 3b).

**Fig. 4:**
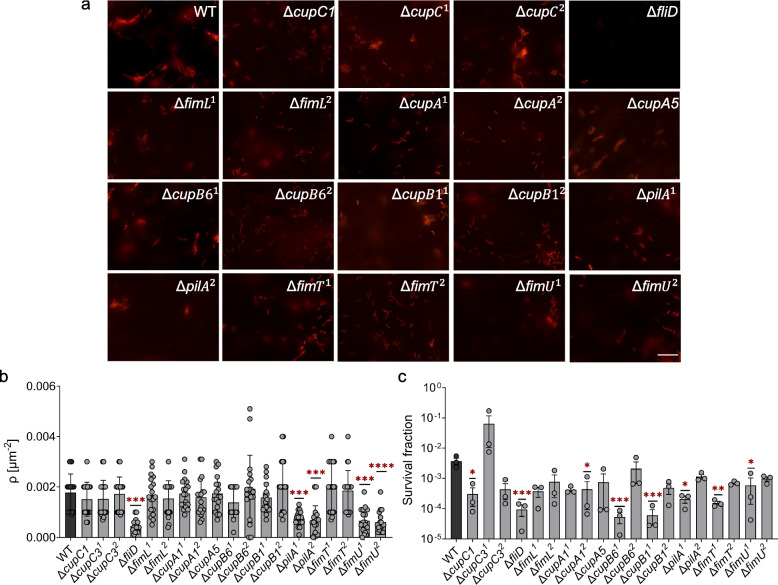
The impact of membrane proteins on the adhesion of *P. aeruginosa*. **a** Micrographs depicting wild-type (WT) as well as mutant *P. aeruginosa* strains on lung cells, with a scale bar of 30 μm. **b** The surface density (*ρ*) of wild-type and the mutant *P. aeruginosa* strains on lung cells. The data presented in this panel correspond to at least three biological replicates, with multiple images captured in each replicate to ensure that diverse populations were included. **c** Bacterial cell survival fractions. Bacterial cells were cocultured with lung cells for 2 h at 37 °C, followed by exposure to ofloxacin (10 × MIC) for 3 h. Clonogenic survival assays were conducted before and after treatments to quantify the surviving cell fractions. One-way ANOVA with Dunnett’s post-test was utilized for pairwise comparisons, where *P < 0.05, **P < 0.01, ***P < 0.001, ****P < 0.0001. The *F*-statistics were used for the correlation analysis. The data for each time point is represented as the mean value ± standard deviation. *N* = 3

The displacement distribution that appears more homogeneous and normal in Δ*fliD* suggests that these cells within the population are demonstrating consistent behavior and predictable motility (Fig. 3b). Given that adhesive properties influencing cell interactions with surfaces or other cells may play a crucial role in shaping the observed displacement patterns, the more normal distribution in Δ*fliD* cells suggests they might be less adhesive compared to the wild type or other mutants. To test this, we subjected both the wild-type and mutant strains of *P. aeruginosa*, with equal cell numbers, to lung cell-coated surfaces for an extended period (2 h) to quantify adhered cells (i.e., *ρ* values) (Additional file 1: Table S3). Although the Δ*fliD* strain exhibited a decreased number of cells adhered to the lung cell surface compared to the *P. aeruginosa* wild type, as expected (*P* < 0.0001, ANOVA with Dunnett’s post-test, Fig. 4a, b), this reduction was also observed in Δ*pilA*^1^, Δ*pilA*^2^, Δ*fimU*^1^, and Δ*fimU*^2^ strains, indicating that cellular adherence properties are more complex and cannot be defined by motion alone; other cellular proteins also play a key role. To confirm these results and further investigate the consistency of the findings, the experiment was repeated using the skin cell line. Micrographs reveal that the Δ*fliD*, Δ*pilA*^1^, Δ*pilA*^2^, Δ*fimU*^1^, and Δ*fimU*^2^ mutants adhered less to skin cells than the wild-type strain (*P* < 0.01, ANOVA with Dunnett’s post-test, Additional file 1: Fig. S4a, b), which is consistent with the observations in lung cell cultures (Fig. 4a, b). Moreover, the changes in cell adhesion observed in the mutant strains are independent of their growth, as there were no notable variations in the total number of cells (both swimming and adhered cells) observed between the mutant strains within a 2-h time frame (Additional file 1: Fig. S5a, b).

### The mutant strain Δ*fliD* demonstrates a reduced survival fraction

Bacterial adhesion represents a significant health concern, as it can lead to the development of infections that are notoriously challenging to treat. This is largely because cells growing in dense bacterial layers on host surfaces [38] can exhibit an exceptional capacity to survive lethal doses of antibiotics [39]. To investigate how the adhesin molecules facilitating interactions impact antibiotic-mediated killing, both wild-type and mutant strains of *P. aeruginosa* were exposed to lung cell-coated surfaces for 2 h and then treated with ofloxacin (an effective antibiotic for *P. aeruginosa*) at 10 × minimum inhibitory concentration (MIC). We performed a 3-h treatment, which resulted in a biphasic kill curve in the wild-type strain, and quantified survival fractions by calculating the ratio of colony-forming units after the 3-h treatment to the initial number of cells (Additional file 1: Fig. 5c). The MICs of the mutant strains were not significantly different from that of the wild-type strain (Additional file 1: Table S4), indicating that the mutant strains did not acquire any resistance. Bacterial cells were collected before and after treatment from both the supernatant and mammalian-cell surfaces to conduct clonogenic survival assays. It should be noted that adhesion and antibiotic survival mechanisms are diverse and complex, and there may be distinct mechanisms that do not overlap; however, our results revealed the presence of overlapping mechanisms involving *fliD* gene whose deletion led to decreased adherence and antibiotic survival (Fig. 4c), underscoring its therapeutic potential. In addition to the Δ*fliD* strain, interestingly, nearly all mutant strains with genetically perturbed membrane proteins (except Δ*cupC3*^*1*^) showed varying degrees of reduced survival fractions compared to the wild type (Fig. 4c). Specifically, mutants like Δ*cupB6*^*1*^, Δ*cupB1*^*1*^, and Δ*fimT*^*1*^ exhibited significantly lower survival levels (Fig. 4c). We would like to note that these mutant strains involve transposon insertions (rather than single gene knockouts), which can potentially alter the upstream or downstream genetic structures of the affected genes. This may explain the variation observed between strains with the same gene perturbation, such as Δ*cupC3*^*1*^ vs. Δ*cupC3*^*2*^ (Fig. 4c).

Given that bacterial aggregation can create a protective environment for the bacteria and can trigger a broad-spectrum antimicrobial response [40, 41], we also compared the survival fractions of bacterial strains in the presence or absence of mammalian cells under identical conditions. We observed that some *P. aeruginosa* strains, including the wild type, showed significantly increased survival fractions in the presence of mammalian cells compared to their survival in the absence of mammalian cells (Additional file 1: Fig. S6). However, the survival of some strains, including Δ*fliD*, was not affected by the presence of mammalian cells (Additional file 1: Fig. S6), suggesting that, in addition to adhesive properties, genetic variations or additional cellular proteins also play a role in antibiotic survival.

## Discussion

The data presented in this study was collected from our experiments, in which we conducted imaging near the top surface of the mammalian cells at a minimum frame rate of 22 fps for about 20 s (the “Methods” section). These short movies with multiple biological replicates allowed us to monitor 5600–22,005 trajectories (depending on the strains), providing us with a good representation of population behaviors. These short-term interactions offer valuable insights into the long-term adhesion of bacteria to surfaces, as it appears that short-term interactions exert influence on long-term outcomes. *P. aeruginosa* cells exhibited lower MSD values compared to *E. coli*, indicating more constrained motion and greater adhesion to mammalian cells. This constrained motion is likely a result of *P. aeruginosa*’s ability to adhere more effectively to surfaces, which limits its movement and results in shorter trajectories. The higher adhesion capability of *P. aeruginosa* is significant, as it enhances the bacterium’s ability to form biofilms and establish infections on host tissues. *E. coli*, in contrast, displayed longer trajectories and higher MSD slopes, reflecting less frequent adherence. The higher MSD values and longer trajectories suggest that *E. coli* cells are more motile and less likely to attach to surfaces, allowing them to move more freely in their environment. This difference in behavior between the two species could be attributed to the distinct structural and functional characteristics of their surface proteins, such as pili and flagella, which play crucial roles in mediating adhesion and motility [42].

When analyzing MSD vs. time interval plots, the expectation is usually a monotonically increasing curve (Fig. 1i, l, and Additional file 1: Fig. S3). However, MSD values might sometimes increase and then decrease with respect to time intervals due to several factors. Cells might encounter physical barriers or confinements in their environment, initially moving freely and increasing the MSD, but then becoming restricted, which leads to a decrease in MSD. Additionally, cells might exhibit cyclic or periodic behaviors, such as oscillations or back-and-forth movements, resulting in an initial MSD increase followed by a decrease as the cell returns to its starting point or an equilibrium position. These explanations are supported by our movies showing bacterial and mammalian cell interactions (Additional file 1: Fig. S1 and Additional file 2: Movie S1).

Analyzing the mutant strains of *P. aeruginosa* with perturbations in key membrane proteins showed that Δ*fliD* exhibited a more homogeneous and normal (Gaussian-like) displacement distribution, unlike wild-type *P. aeruginosa* and *E. coli* cells. A cell displacement distribution that appears more homogeneous and normal provides significant insights into cellular behavior and environmental interactions. Homogeneity suggests that despite genetic perturbations affecting motility, the cells within the population exhibit consistent movement patterns, possibly relying on diffusive characteristics. The reduced adhesion of Δ*fliD* cells also highlight the interplay between motility and adhesion in influencing bacterial behavior and interactions with their environment. The *fliD* gene codes for a subunit of the FliD ring complex, which is part of the flagellar apparatus in bacteria and essential for the proper assembly of the flagellum[43, 44]. The flagellum is a long, slender filament that functions as a whip-like organelle, allowing the bacterium to move [37, 43, 44]. The flagella contribute to adhesion through two mechanisms: swimming motility and entrapment of flagella within microstructures. We think that the decrease in adhesion results from a lower rate of collision with the surface and reduced hydrodynamic interaction, as the bacteria can only approach the surface through Brownian motion. Furthermore, flagella seem to play a role in retaining bacteria within submicron structures on the surface [45]. This observation is consistent with a previous study, which reported reduced adhesion on the smooth surface of hexadecane droplets in the absence of motility to become trapped at the interface [5]. In cystic fibrosis, *P. aeruginosa* is known to colonize and is responsible to form biofilm in the airways which in general is intrinsically tolerant to multiple antibiotics[46]. Deletion of the *flgE* gene associated with the flagellum has been shown to increase the formation of microcolony aggregates in a microfluidic environment and developed multidrug tolerance in biofilms[46]. However, cells harboring *fliD* mutation had significant attachment deficiency on the mucin-coated surface[47], which is, indeed, consistent with our results.

Along with the Δ*fliD* strain, the mutant strains Δ*pilA* and Δ*fimU* exhibited reduced adhesion, suggesting that the adhesion properties of *P. aeruginosa* are complex and multifaceted, involving various genetic components. In *P. aeruginosa*, pili play a crucial role in twitching motility[17]. The *pilA* gene is essential for the formation of pilus rods and the tip of pilin (PilA) can bind to biotic and abiotic surfaces[17]. PilA serves as a grappling hook that enables the bacterium to move along the surface to which it is adhered[35]. Our study showed that a mutation in the *pilA* gene led to significantly reduced adhesion to mammalian cells compared to the wild-type strain (Fig. 4b). It has been well-documented in the literature that mutations in this gene can lead to a reduction in the virulence of *P. aeruginosa*[22, 48]. Comparisons of various mutants’ abilities to associate with and invade corneal epithelial cells have revealed that the nonpiliated *pilA* mutant demonstrated reduced adherence and invasion, highlighting its importance in virulence[22]. While *pilT* and *pilU* mutants, which also have impaired twitching motility, may display decreased cytotoxicity towards epithelial cells in vitro, they did not exhibit substantial changes in adherence to corneal epithelial cells when compared to the wild-type strain[22]. The Δ*fimT* mutant, which is responsible for a minor pilin that can also contribute to twitching motility[36], did not exhibit a significant decrease in adhesion to mammalian cells in our study. In contrast, the Δ*fimU* mutant showed a lower level of adhesion compared to the Δ*fimT* mutant (Fig. 4b).

The increased survival fractions of certain *P. aeruginosa* strains, including the wild type, in the presence of mammalian cells indicate that adhesion to host cells may provide a protective advantage, possibly by creating a physical barrier or by fostering a more favorable microenvironment for bacterial survival. In the context of infection sites, bacteria typically undergo aggregated growth, resulting in the development of antibiotic-tolerant traits[49–52]. These aggregates are primarily formed through the “depletion aggregation” mechanism facilitated by extracellular substances (e.g., host polymers and surface proteins), causing antibiotics to be less effective [49, 50]. Bacterial cells aggregate through “bridging aggregation,” a process driven by adhesions and interactions between the extracellular substances and bacteria, contributing to antibiotic tolerance[50]. Bacterial aggregation at infection sites enhances antibiotic tolerance [49–52], and our study’s results further corroborate this phenomenon. However, the fact that some strains did not exhibit increased survival in the presence of mammalian cells implies that other factors, such as specific genetic variations or the expression of additional cellular proteins, also influence bacterial adherence and survival. This underscores the complexity of bacterial adhesion and tolerance and suggests that a multifaceted approach is needed to fully understand and combat bacterial infections.

## Conclusions

Our method of categorizing bacterial cell trajectories based on their motion can pinpoint key mechanisms crucial for the adhesion and tolerance properties of bacterial cells. These two properties contribute to the development of persistent infections that are especially difficult to treat. We believe that our strategy is transformative, as it offers an effective method for investigating the complex nature of bacterial cell behaviors in a highly heterogeneous population, which can be easily adapted to other bacterial species and cell lines.

## Methods

### Bacterial strains and plasmids

*Escherichia coli* K-12 MG1655 wild-type, *Pseudomonas aeruginosa* (PAO1), and various mutant strains of PAO1 were used for the experiments. The PAO1 mutants were obtained from Manoil Lab at the University of Washington, Seattle. The mutant strains were confirmed through PCR using internal and external check primers. To visualize the bacteria under the microscope, we used an isopropyl β-d-1-thiogalactopyranoside (IPTG) inducible pMMB67EH-*mCherry* expression system. This system was constructed using a cloning technique [53] from plasmid pMMB67EH-*yfp* [54], which was purchased from Addgene (Catalog# 90104). Additional file 1: Table S1 presents a complete list of the strains and plasmids used in the study as well as the cloning and the PCR check primers.

### Bacterial culture conditions

All chemicals used were purchased from ThermoFisher Scientific (Waltham, MA), VWR International (Radnor, PA), or Sigma-Aldrich (St. Louis, MO) unless otherwise stated. The bacterial strain *E. coli* was cultured in Luria–Bertani (LB) broth and LB agar plates. LB broth was prepared by mixing 5.0 g of yeast extract, 10.0 g of tryptone, and 10.0 g of sodium chloride in 1 L of deionized water, while LB agar plates were made by mixing 10.0 g of tryptone, 10.0 g of sodium chloride, 5.0 g of yeast extract, and 15.0 g of agar in 1 L of deionized water. To culture *P. aeruginosa* in liquid media, Mueller–Hinton (MH) broth was used, which was prepared by mixing 2.0 g of beef extract powder, 17.5 g of acid digest of casein, and 1.5 g of soluble starch in 1 L of deionized water. For determining the colony forming units of *P. aeruginosa*, MH agar plates were used, which were prepared by mixing 2.0 g of beef extract powder, 17.5 g of acid digest of casein, 1.5 g of soluble starch, and 17.0 g of agar in 1 L of deionized water. Phosphate-buffered saline (PBS) solution (1X: 1.37 M NaCl, 0.027 M KCl, and 0.119 M phosphates) was used to wash the bacterial cells. Whenever needed, 25 μg/ml gentamicin was used to maintain the plasmids in the bacteria. Isopropyl β-d-1-thiogalactopyranoside (IPTG) at 1 mM was added to the cultures to express mCherry in the bacteria. Ofloxacin at 10 μg/ml (10 × MIC) was used in the clonogenic survival assays. To create a stock solution of ofloxacin, 5 mg of ofloxacin was mixed with 1 ml of deionized water and then titrated with 1 M NaOH until the solution was fully dissolved. The minimum inhibitory concentration of ofloxacin was measured using a commercially available Etest strip (Catalog# 22–777-878, Fisher Scientific) (Additional file 1: Table S2). All the chemicals soluble in deionized water were sterilized with a 0.2-μm syringe filter. To sterilize the liquid and solid media, an autoclave was used (121 °C, 30 min). The chemicals used in this experiment, including their vendors, catalogs, and purity, are tabulated in Additional file 1: Table S3.

For bacterial cell culture experiments, *E. coli* and *P. aeruginosa* cells (harboring *mCherry* expression systems) from 25% glycerol cell stocks that had been stored at − 80 °C were streaked on LB and MH agar plates, respectively. Then, the plates were left to grow for 16 h at 37 °C. Subsequently, overnight cultures were generated by inoculating a single colony obtained from the streaked plates in 2 mL of LB or MH broth in test tubes that were supplemented with 25 μg/mL gentamicin (to retain *mCherry* expression plasmids) and 1 mM IPTG (to express *mCherry*). The cultures were then grown in a shaker at 37 °C and 250 rpm for 18 h. To prepare subcultures, overnight cultures were diluted by 100 folds in 2 mL of LB or MH broth in test tubes and then incubated at 37 °C and 250 rpm for 5.5 h.

### Mammalian cell lines and culture conditions

Skin (A375) and lung (H1975) cancer cell lines were purchased from American Type Culture Collection (Manassas, VA). The cells were cultured in Dulbecco’s Modified Eagle Medium/Nutrient Mixture F-12 (DMEM/F-12) supplemented with 10% fetal bovine serum, 100 units of penicillin, and 100 μg/mL streptomycin. They were maintained in a T75 flask in a humidified incubator at 37 °C and 5% CO_2_. To transfer the cells to 6-well plates, the cells in the T75 flasks, at an early passage (passage number 4–6), were washed twice with 10 mL of Dulbecco’s phosphate-buffered saline (DPBS) and then detached from the flasks with 2 mL of trypsin-ethylenediaminetetraacetic acid (EDTA) consisting of 0.25% trypsin and 0.9 mM EDTA for approximately 1–2 min. Then, 5 mL of DMEM/F-12 was added, and the cell suspension was transferred to a 10-mL centrifuge tube. The cell suspension was centrifuged at 800 rpm for 5 min, and the supernatants were removed. The cell pellets were resuspended in fresh drug-free media and transferred to 6-well plates with a square (23 mm × 23 mm) glass coverslip (Catalog# 470,145–876, VWR) placed at the bottom of each well. The glass coverslips were sterilized by washing in 70% ethanol, followed by deionized water, and a 2-h UV sterilization in a biosafety cabinet. The number of cells in each well was 2 million (M) for A375 cells and 1.5 M for H1975. These seeding concentrations of mammalian cells, allowed to grow for 2 days, achieved 100% confluency on the small coverslips. To determine these optimal concentrations, cell confluency assays were conducted with varying seeding densities, and we used phase contrast images to confirm complete coverage of mammalian cells on the coverslips. Subsequently, to ensure cell viability and membrane integrity, DAPI and LIVE/DEAD staining were employed (details below). Each well contained 2.5 mL of DMEM/F-12 medium and was incubated at 37 °C and 5% CO_2_ for 2 days. For the clonogenic survival assay, cells were grown without glass coverslips.

### Nuclear and live/dead staining

If necessary, after transferring cells to 6-well plates with coverslips, staining was performed for nuclear and live/dead cells using DAPI (4′,6-diamidino-2-phenylindole) (Catalog# D1306, ThermoFisher Scientific) and the ReadyProbes Cell Viability Imaging Kit (Catalog# R37609, ThermoFisher Scientific), respectively, following the protocols provided by the vendors. Fluorescence quantification of the stained cells was conducted in the standard blue fluorescence channel (excitation: 360 nm and emission: 460 nm) and the green fluorescence channel (excitation: 470 nm and emission: 525 nm) using the EVOS M7000 fluorescence microscopy (Catalog# AMF7000, ThermoFisher Scientific). The blue DAPI reagent is able to penetrate cells and stains the DNA in the nucleus. The green dead cell reagent is a cell impermeable, so dead cells emit green fluorescence. Live and dead cells were used as controls, with dead cells generated by treating them with 70% ethanol for 30 min.

### Infecting mammalian cells with *bacteria*

To prepare bacterial subcultures, overnight cultures of *Pseudomonas aeruginosa* carrying *mCherry* expression plasmids were diluted 100-fold in 2 mL fresh MH media in 14-mL test tubes and then grown at 37 °C in a shaker at 250 rpm. Gentamicin (25 μg/mL) was added to the cultures to preserve the plasmid, while a concentration of 1 mM IPTG was maintained in the bacterial cultures to induce the expression of the red fluorescent protein (mCherry). At the early stationary phase (*t* = 5.5 h), the bacteria subcultures were centrifuged at 4800 rpm for 10 min to remove the medium and then resuspended in DMEM/F-12 supplemented with 50 mM HEPES to obtain a desired bacterial concentration that corresponds to the desired cell concentration of 5, 10, 25, 50, 75, and 100 million (M) cells/mL. The cell concentration was determined by flow cytometry. To prepare for the infection, mammalian cell cultures in 6-well plates with coverslips were washed twice with 1 mL DPBS, and then 1 mL of fresh DMEM/F-12 supplemented with 50 mM N-2-hydroxyethylpiperazine-N′-2-ethanesulfonic acid (HEPES) was added to each well. The mammalian cells were infected with bacteria by removing the medium from and adding 2 mL of the bacteria suspension into each well, followed by incubation at 37 °C for 2 h. Different durations of incubation were also evaluated as detailed below.

### Surface density of *bacteria* on mammalian cells as a function of bacterial cell concentrations

To infect mammalian cells (A375 or H1975), bacteria at concentrations (*E. coli* or *P. aeruginosa*) of 5, 10, 25, 50, 75, and 100 M/mL were added to each well of 6- well plate having mammalian cells on a coverslip and incubated at 37 °C for 2 h, as described above. The liquid media from each well was subsequently removed, and the wells were washed three times with DPBS and once with 1 mL of DMEM/F-12 to completely remove unattached bacteria. Then, the coverslips with mammalian cells and adhered bacteria were carefully removed from the wells for imaging. A microscope glass slide with an imaging spacer (Catalog# 102,096–614, VWR) was used and the coverslips were effectively sealed with vacuum grease (Catalog# 044224-KT, Dow Corning). Using a fluorescence microscope (EVOS FL Auto 2, Invitrogen), at least 7 images per slide were acquired using a 100 × oil immersion objective lens (Olympus, catalog no. AMEP4733; working distance, 0.3 mm). Images were taken under brightfield, red channel (excitation: 585 nm and emission: 611 nm), and blue channel (excitation/emission: 360/460) and analyzed with the ImageJ software. The “cell counter” function of ImageJ was used to quantify the adhered cells. Surface density (*ρ*) on the surface was calculated by normalizing the number of bacteria on the surface with the surface area.

### The surface density of *bacteria* on mammalian cells as a function of time

To determine the density of bacteria on the mammalian cell surface, mammalian cells were infected with bacteria (10 M/mL) and incubated at 37 °C for various durations including 0, 10, 20, 50, 85, and 120 min, as described above. At *t* = 0 min, the bacteria suspension was immediately removed from the well. At each time point, the liquid media was removed, and the cells were washed with DPBS three times, followed by one wash with 1 mL of DMEM/F-12 to remove unattached bacteria. Microscope slides were prepared and imaged as described in the previous section. The number of bacterial cells on the surface was quantified using the ImageJ software. The number of bacteria on the surface was normalized with the surface area to calculate surface density, *ρ*. For *P. aeruginosa* PAO1 mutant strains, mammalian cells were infected with each mutant (10 M/mL) and incubated for 2 h. Microscope slide preparation, imaging, and the quantification of bacterial surface density were performed as described above.

### Tracking *bacteria* near surface of mammalian cells-coated surface

In order to study the cell dynamics near the surface of lung cells (H1975), mammalian cells were cultured in a 6-well plate, as described in the previous section. Lung cells were selected for this experiment because *P. aeruginosa* is highly pathogenic in the lungs of cystic fibrosis patients, and this cell line provides a more homogeneous coverage of the glass surface than skin cells (A375). *P. aeruginosa* PAO1 and mutant strains (Additional file 1: Table S1) were selected for the experiment, with the wild-type strain used as a control. Bacterial subcultures were centrifuged at 4800 rpm for 10 min, and the supernatants were discarded. The pellets were resuspended in DPBS and diluted with DPBS to obtain a cell concentration of 10 M/mL. DPBS was used to reduce the photobleaching of samples. An imaging spacer was used to prepare a microscope slide, and 18 μL of bacterial suspension was added to the slide. The slide was then sealed with a mammalian cell-coated glass coverslip, and vacuum grease was used to seal the slide further. Imaging (red channel) was conducted near the top surface of the mammalian cell at a minimum frame rate of 22 fps for at least 20 s, and at least three movies were acquired for each slide. The movement of swimming and attached cells was analyzed as described in the following section, and the mean square displacement (MSD) was calculated and reported.

MSD is a measurement of the square of the distance traveled by a particle over time and is defined as follows:

$$MSD= \langle {(x\left(t\right)-{x}_{0})}^{2}\rangle +\langle {(y\left(t\right)-{y}_{0})}^{2}\rangle =4Dt$$

where $(x\left(t\right), y(t))$ is position of a cell at time $t$, ${(x}_{0},{y}_{0})$ is the initial position of cell at $t=0$, and $D$ is the diffusivity of cell.

A minimum of three biological replicates were performed. The movies were captured using EVOS FL Auto 2 fluorescence microscopes with precision to prevent any biased results. Adjustments were made to the microscope’s exposure time, gain, and lighting to minimize pseudo cells presence in the videos and ensure a dark background. The movies were analyzed using a single cell tracking algorithm written in MATLAB and a criterion was set to distinguish cells attached from swimming or free-floating cells [5]. A filter parameter effectively eliminated any residual background noise during the video analysis process. Additionally, an appropriate threshold value, slightly larger than the size of a cell in pixels, was employed for cell identification. Finally, stray noises arising during the adjustment of video contrast were eliminated by applying an optimized threshold value to the filter parameter. All these parameters were kept constant for every movie for consistency in the analysis. Once the cell tracking was completed, the MATLAB program provided us with the x and y displacement of each tracked cell along the lag time and cell ID, which were used to calculate the MSDs and to generate the trajectory figures (details regarding the tracking algorithms can be found in the following studies [5]). Displacement of cells can vary with time. We use an average displacement of each cell per frame for its entire trajectory length to define the cell as attached or unattached, irrespective of cell’s stop-swim motion. Cells with adhered cell trajectories that moved less than one pixel in one frame were considered attached. MSD was calculated for both swimming and attached cells, and 5600–22,005 tracks (depending on the strains, Fig. 3) were used to calculate MSDs for all the strains.

### Generating histograms

For each movie acquired at 22 fps, we calculated the displacement of each cell (i) in each frame (Δt = 1/22 s). We then determined the average displacement (<Δr_i_ >) for each cell by averaging its displacement [Δr_i_(Δt)] over the entire track length (*n*).

Displacement for each cell at time interval (Δt) was computed using the Euclidean distance formula:

$$\Delta {r}_{i,j}\left(\Delta t\right)=\sqrt{{\left({x}_{i}\left(j+\Delta t\right)-{x}_{i}\left(j\right)\right)}^{2}+{\left({y}_{i}\left(j+\Delta t\right)-{y}_{i}\left(j\right)\right)}^{2}}$$

This calculation was performed for the entire length of cell trajectory (*l*_*i*_), generating a series of displacements for each cell. Hence, the average displacement is given by,

$$\langle \Delta {r}_{i}\rangle =\frac{1}{{l}_{i}}\sum_{j=0}^{{l}_{i}-\Delta t}\Delta {r}_{i,j}\left(\Delta t\right)$$

where *l*_*i*_ is the total number of time intervals for cell *i*.

The average displacements of all cells were compiled into a single dataset, representing the overall displacement behavior of the cell population. The range of average displacements was divided into bins of equal width, based on the distribution of average displacements to ensure adequate representation. Histograms with a bin size of 0.1 μm were generated to show how displacement is distributed within the entire subpopulation. The *y*-axis of the histogram represents the percentage of the population, normalizing the number of cells in each bin with the total number of cells.

### Clonogenic survival assays

To prepare for co-culturing with lung cells, overnight cultures of *Pseudomonas aeruginosa* harboring *mCherry* expression plasmids were diluted 100-fold into 2 mL fresh MH media in 14-mL test tubes and grown in a shaker at 250 rpm at 37 °C. Gentamicin (25 μg/mL) was added to the cultures to maintain the plasmid, while 1 mM IPTG was maintained to promote expression of mCherry. At the early stationary phase (*t* = 5.5 h), the bacterial cells were collected and washed once by centrifugation at 4800 rpm for 10 min. The pelleted cells were resuspended in DMEM/F-12 media with 50 mM HEPES to reach a cell concentration of 10 M/mL and were then used to infect the lung cells in a 6-well plate format. After incubating infected mammalian cells at 37 °C for 2 h, the cells were treated with ofloxacin (10 × MIC, 10 μg/mL) for 3 h. Following the treatment, the supernatant of the co-cultures was collected and washed twice with DPBS (1X) by centrifugation at 17,000 rpm for 3 min. To collect bacterial cells that adhered to the mammalian cells, cells were trypsinized and then collected and washed twice with DPBS immediately by centrifugation to remove the antibiotics. After the final centrifugation, 900 μL supernatant was removed, and the cell pellets were resuspended in the remaining 100 μL of DPBS. Both resuspended cell samples obtained from supernatant and mammalian cell surfaces were serially diluted in DPBS in a tenfold manner and then spotted on a MH agar plate to count the colony-forming units. The agar plates were then incubated for 16 h at 37 °C for the colonization of the surviving cells. Similarly, cells were plated on an agar plate before and after the initial 2-h incubation period to determine the initial number of bacterial cells. These procedures were repeated under identical conditions without mammalian cells to quantify bacterial cell survival fractions in the absence of mammalian cell interactions.

### Statistical analysis

Unless otherwise stated, a minimum of three biological replicates were conducted for each experiment. In the microscopy analysis, multiple images were acquired and evaluated for each biological replicate to monitor a total of 5600–22,005 (depending on the strains) trajectories. The data are presented as mean ± standard deviation or error. To perform pairwise comparison, a two-way ANOVA with Dunnett’s posttest was utilized. The Kolmogorov–Smirnov test was used to compare the empirical cumulative distribution functions of two histograms. The Kolmogorov–Smirnov test is non-parametric and does not assume a specific distribution shape, which makes it suitable for comparing distributions typically observed in biological data. The statistical analysis was carried out using GraphPad Prism V 9.5.1, and *P*-values are indicated on the plots as follows: * for *P* < 0.05, ** for *P* < 0.01, *** for *P* < 0.001, and **** for *P* < 0.0001.

## Supplementary Information

Additional file 1. Fig. S1. Bacterial cell trajectories. Fig. S2. The changes in the density of bacteria on mammalian cell surfaces in relation to the initial concentration of bacterial cells and co-culturing time. Fig. S3. Individual MSDs of P. aeruginosa strain on lung-cell surfaces. Fig. S4. The impact of membrane proteins on the adhesion and tolerance of P. aeruginosa. Fig. S5. Clonogenic survival assays. Fig. S6. Clonogenic survival assays. Table S1. Bacterial strains, plasmids, mammalian cell lines, and oligonucleotides used in this study. Table S2. Comparing the distributions of average displacements between PA01 and mutant strains. Table S3. Percentage of attached and unattached/motile cells in P. aeruginosa strains. Table S4. MIC levels of ofloxacin for the strains used in this study. Table S5. Chemicals used in this study.

Additional file 2. Movie S1. Bacterial cell trajectories. Trajectories of P. aeruginosa PA01 near the lung cell for (a) an attached bacterium, (b) a surface-bound bacterium rotating in the clockwise direction, (c) a bacterium moving on the mammalian cell surface, and (d) a bacterium approaching the H1975 cell and abruptly turning away. Fluorescent images depicting bacterial cells were exclusively featured in the movies to ensure enhanced resolution. The mammalian cells in the movies were depicted in Supplementary Figure S1a-d, delineated by blue circles for clarity. Movie S2. Representative movies for bacterial strains. (a-b) Untracked and tracked Escherichia coli MG1655 cells, respectively. Single cell tracking in panel “b”, highlighted in red circles, is performed using a MATLAB code. The tracked video (panel b) is ~8 times slower than its original version (panel a). (c-d) Untracked and tracked P. aeruginosa wild-type (WT) cells, respectively. Single cell tracking in panel “d”, highlighted in red circles, is performed using a MATLAB code. The tracked video (d) is ~8 times slower than its original version (panel c). Movie S3. Representative movie of P. aeruginosa ΔfliD strain. (a-b) Untracked and tracked P. aeruginosa ΔfliD strain, respectively. Single cell tracking in panel “b”, highlighted in red circles, is performed using a MATLAB code. The tracked video is ~8 times slower than its original version (panel a).

## Data Availability

The raw data supporting the conclusions of this article are available in figShare. https://doi.org/10.6084/m9.figshare.27157593.v1 [55]. All other raw data are available from the corresponding author on reasonable request.

## Abbreviations

MSD: Mean square displacement
CF: Cystic fibrosis
CFTR: Cystic fibrosis transmembrane conductance regulator
ANOVA: Analysis of variance
MIC: Minimum inhibitory concentration
IPTG: Isopropyl-ß-D-thiogalactopyranoside
LB: Luria-Bertani
MH: Mueller-Hinton
RPM: Revolution per minute
DPBS: Dulbecco’s phosphate-buffered saline
EDTA: Ethylenediaminetetraacetic acid
DMEM: Dulbecco’s modified eagle medium
UV: Ultraviolet
DAPI: 4′,6-Diamidino-2-phenylindole
HEPES: 2-[4-(2-Hydroxyethyl) piperazin-1-yl] ethanesulfonic acid
PCR: Polymerase chain reaction
