# Supplementary material for: Uncovering bacterial-mammalian cell interactions via single-cell tracking
Source: BMC Biol. 2024 Nov 11;22:256. doi: 10.1186/s12915-024-02056-z (PMC11552363; doi:10.1186/s12915-024-02056-z)
Supplement: Supplementary file 1 — Additional file 1. Fig. S1. Bacterial cell trajectories. Fig. S2. The changes in the density of bacteria on mammalian cell surfaces in relation to the initial concentration of bacterial cells and co-culturing time. Fig. S3. Individual MSDs of P. aeruginosa strain on lung-cell surfaces. Fig. S4. The impact of membrane proteins on the adhesion and tolerance of P. aeruginosa. Fig. S5. Clonogenic survival assays. Fig. S6. Clonogenic survival assays. Table S1. Bacterial strains, plasmids, mammalian cell lines, and oligonucleotides used in this study. Table S2. Comparing the distributions of average displacements between PA01 and mutant strains. Table S3. Percentage of attached and unattached/motile cells in P. aeruginosa strains. Table S4. MIC levels of ofloxacin for the strains used in this study. Table S5. Chemicals used in this study. [file 12915_2024_2056_MOESM1_ESM.docx]

**Supplementary Information**

**Uncovering Bacterial-Mammalian Cell Interactions via Single-Cell Tracking**

**Narendra K. Dewangan^a^, Sayed Golam Mohiuddin^a^, Shayne Sensenbach, Prashant Karki, and Mehmet A. Orman***

William A. Brookshire Department of Chemical and Biomolecular Engineering,

University of Houston, Houston, TX 77204-4004

*Corresponding author: morman@central.uh.edu

^a^Narendra K. Dewangan and Sayed Golam Mohiuddin contributed equally.

**Supplementary Figures**


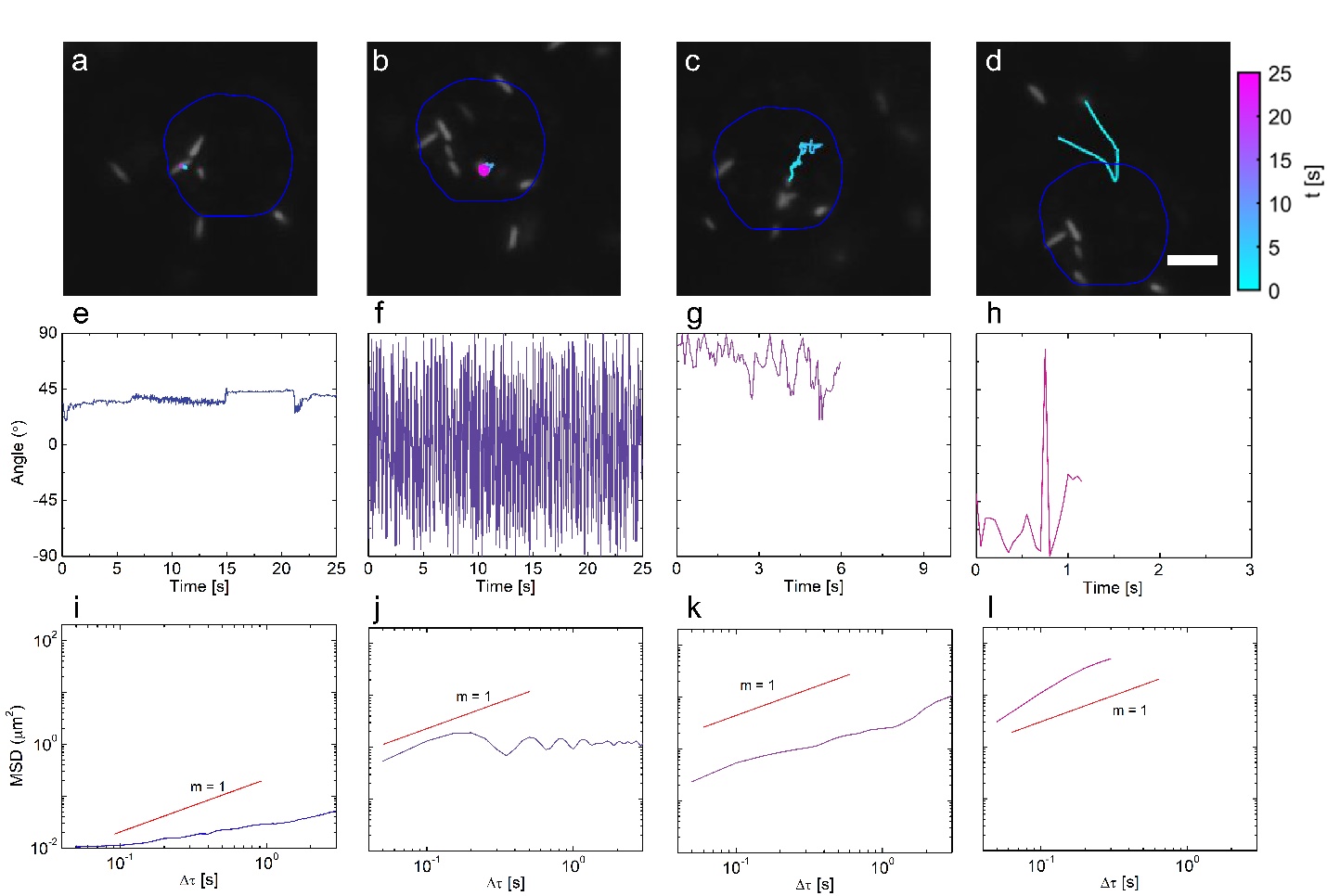
**Fig. S1: Bacterial cell trajectories.** Trajectories of *P. aeruginosa* PAO1 near the lung H1975 cell for (**a**) an adhered bacterium, (**b**) a surface-bound bacterium rotating in the clockwise direction, (**c**) a bacterium moving on the mammalian cell surface, and (**d**) a bacterium approaching the H1975 cell. Scale bar is 5 μm. (**e-h**) Orientation angle of a bacterium as a function of time corresponding to the panels a, b, c, and d, respectively. Note that the bacterium's rotational motion in panel b resulted in an oscillatory angular behavior, as demonstrated in panel f. (**i-l**) Mean square displacements of a bacterium as a function of time corresponding to the panels a, b, c, and d, respectively. The blue circle in panels a, b, c, and d represents the same mammalian cell observed at various times and/or depicted from different angles.


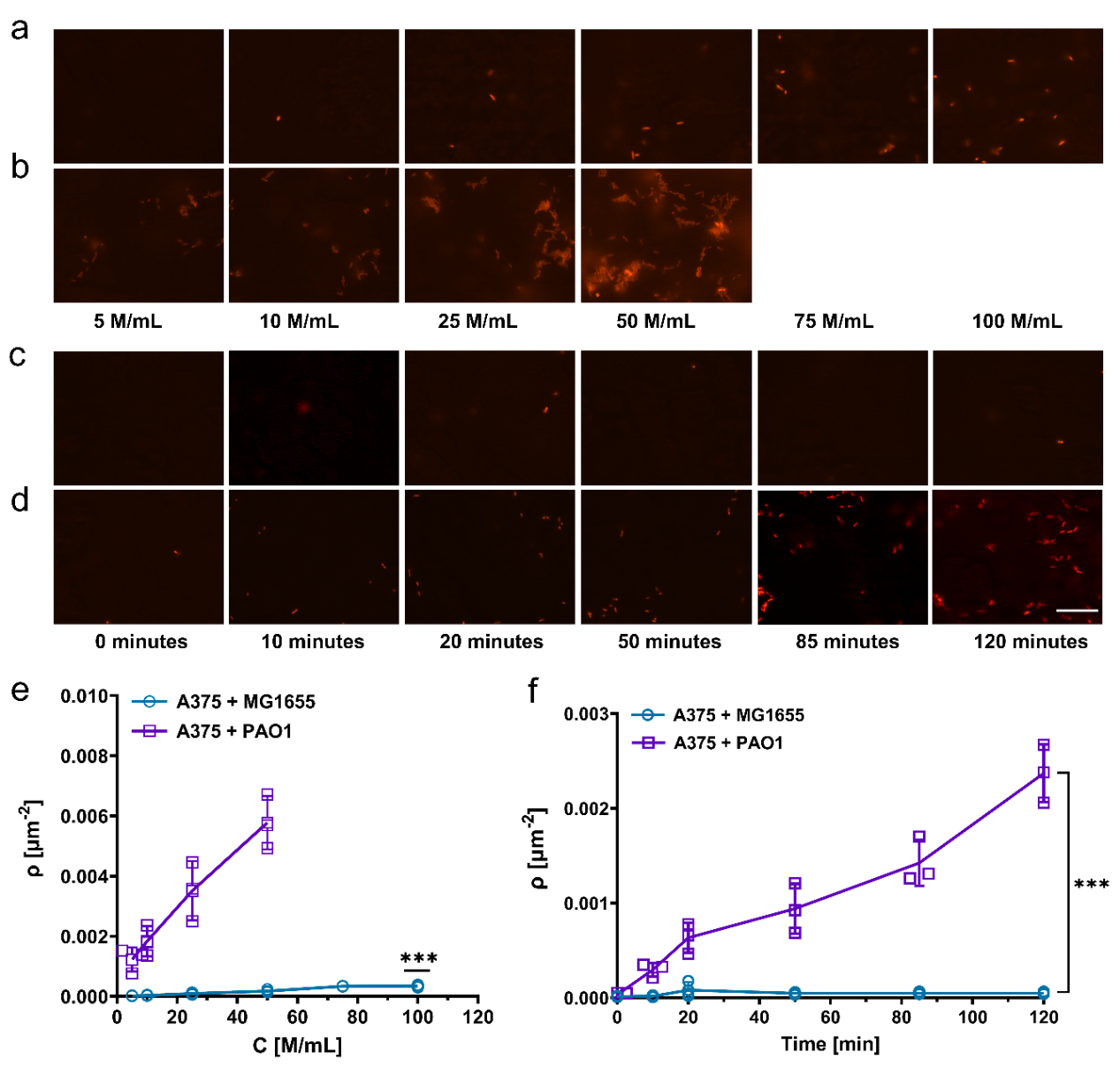


**Fig. S2:** **The changes in the density of bacteria on mammalian cell surfaces in relation to the initial concentration of bacterial cells and co-culturing time.** (**a, c**) Micrographs of *E. coli* MG1655 on skin cells (A375) as a function of the number of bacterial cells (M: million) or co-culturing time, respectively. (**b, d**) Micrographs of *P. aeruginosa* PAO1 on A375 cell line as a function of the number of bacterial cells or co-culturing time, respectively. The scale bar is 30 μm. (**e, f**) The surface density (ρ) of bacteria as a function of the number of bacterial cells or co-culturing time, respectively. The surface density is defined as the number of bacterial cells per unit surface area. The *F*-statistics were used to perform a statistical comparison between two groups, where ***P < 0.001. The data for each time point is represented as the mean value ± standard deviation. N= 3.


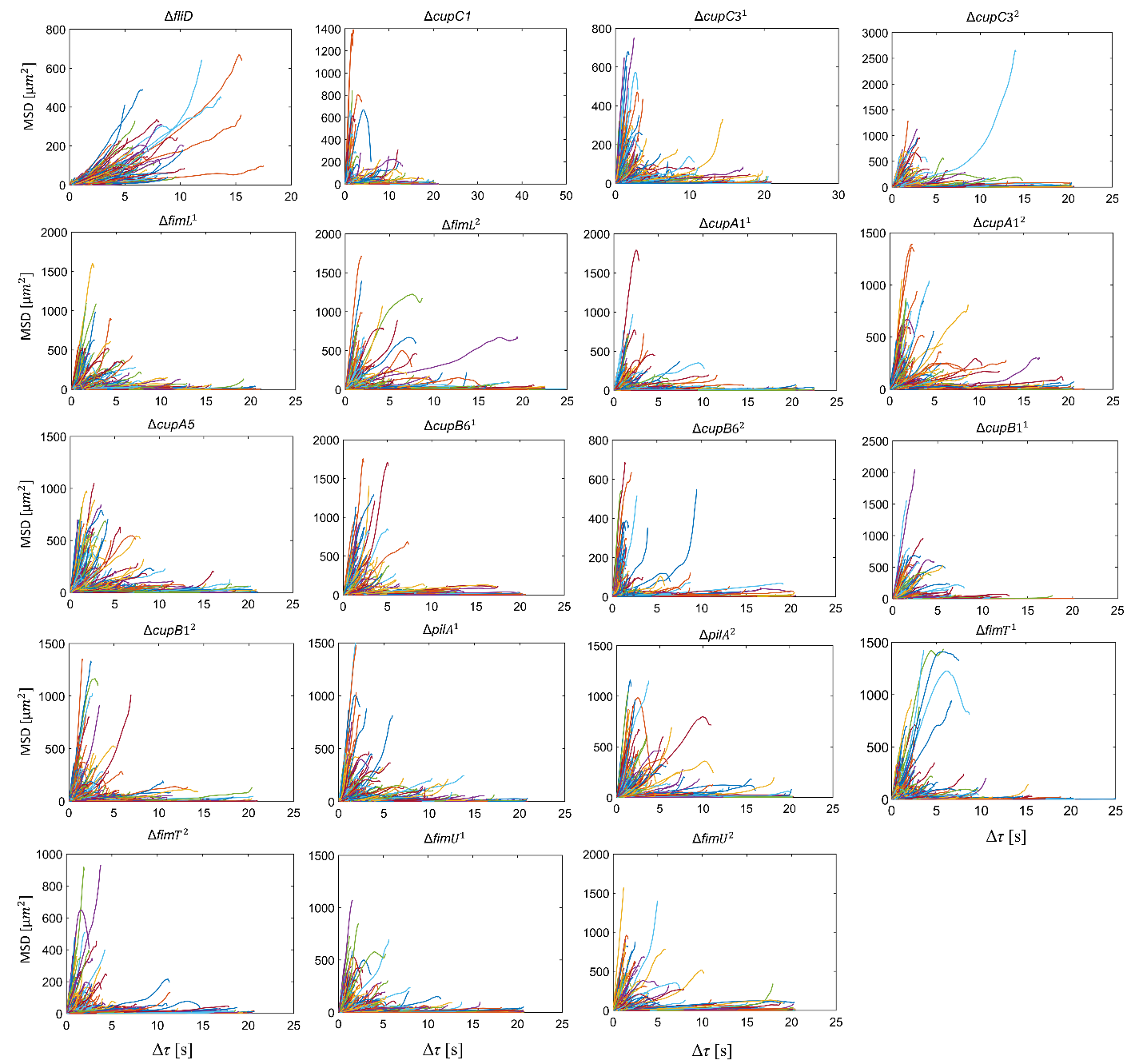


**Fig. S3: Individual** **MSDs of *P. aeruginosa* strains on lung-cell surfaces.** At the population level, the individual MSD of each cell were analyzed for the specified mutants of *P. aeruginosa*. The data presented in each panel correspond to a minimum of three biological replicates, with multiple movies captured in each replicate, ensuring a sufficient number of cell trajectories were analyzed: Δ*fliD*=5,600; Δ*cupC1*=12,553; Δ*cupC3^1^*=12,539; Δ*cupC3^2^*=22,005; Δ*fimL^1^*=15,633; Δ*fimL^2^*=16,440; Δ*cupA1^1^*=14,914; Δ*cupA1^2^*=12,751; Δ*cupA5*=16,040; Δ*cupB6^1^*=12,937; Δ*cupB6^2^*=7375; Δ*cupB1^1^*=7070; Δ*cupB1^2^*=13,414; Δ*pilA^1^*=11,837; Δ*pilA^2^*=9,837; Δ*fimT^1^*=13,607; Δ*fimT^2^*=15,749; Δ*fimU^1^*=7,862; Δ*fimU^2^*=11,402 trajectories.


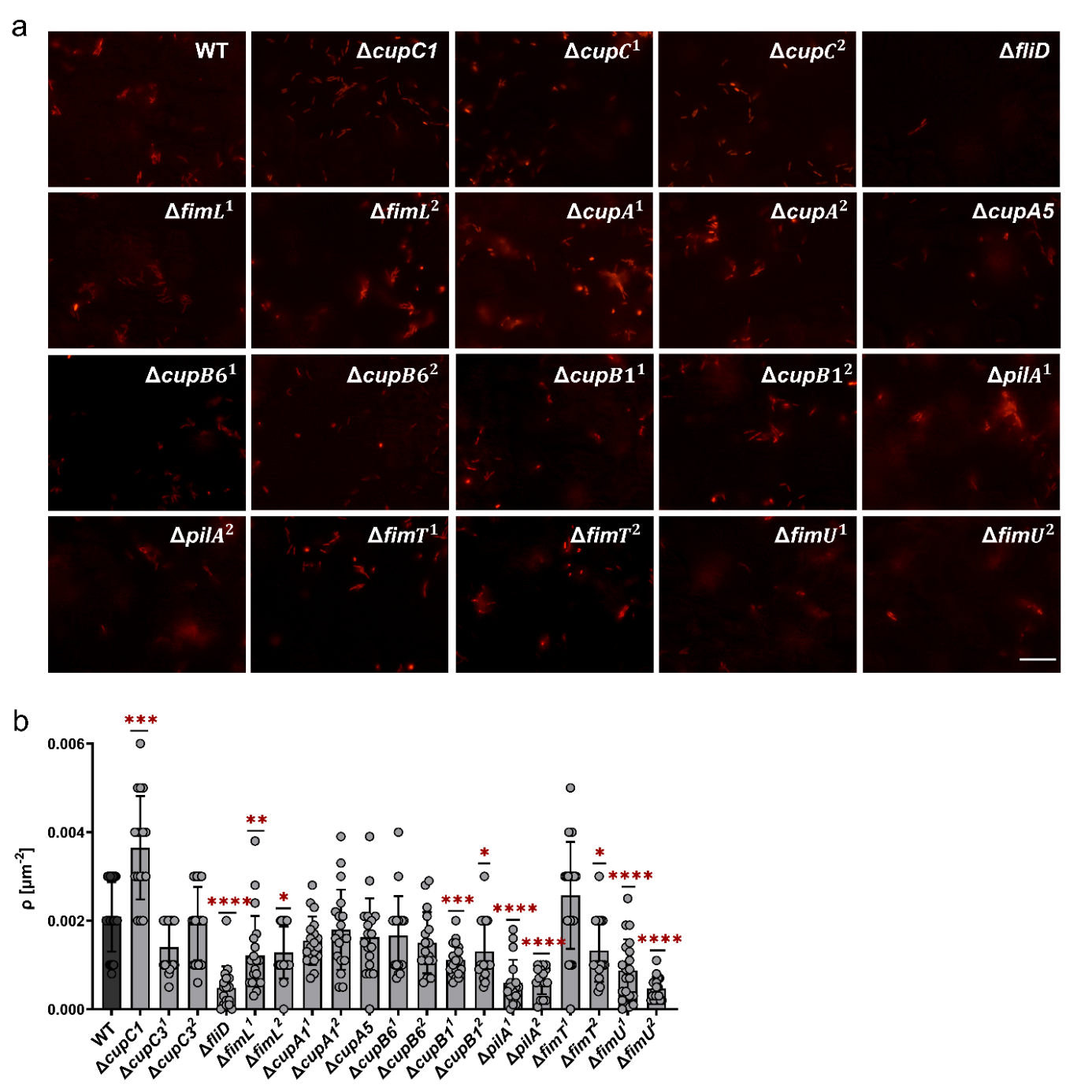


**Fig. S4:** **The impact of membrane proteins on the adhesion and tolerance of *P. aeruginosa*.** (**a**) Micrographs depicting wild-type *P. aeruginosa* as well as mutant strains on skin cells (A375), with a scale bar of 30 μm. (**b**) The surface density (ρ) of wild-type *P. aeruginosa* and the mutant strains on A375 cell line. One-way ANOVA with Dunnett's post-test was utilized for the statistical analysis, where *P < 0.05, ***P < 0.001, ****P < 0.0001. The data for each time point is represented as the mean value ± standard deviation. N=3.


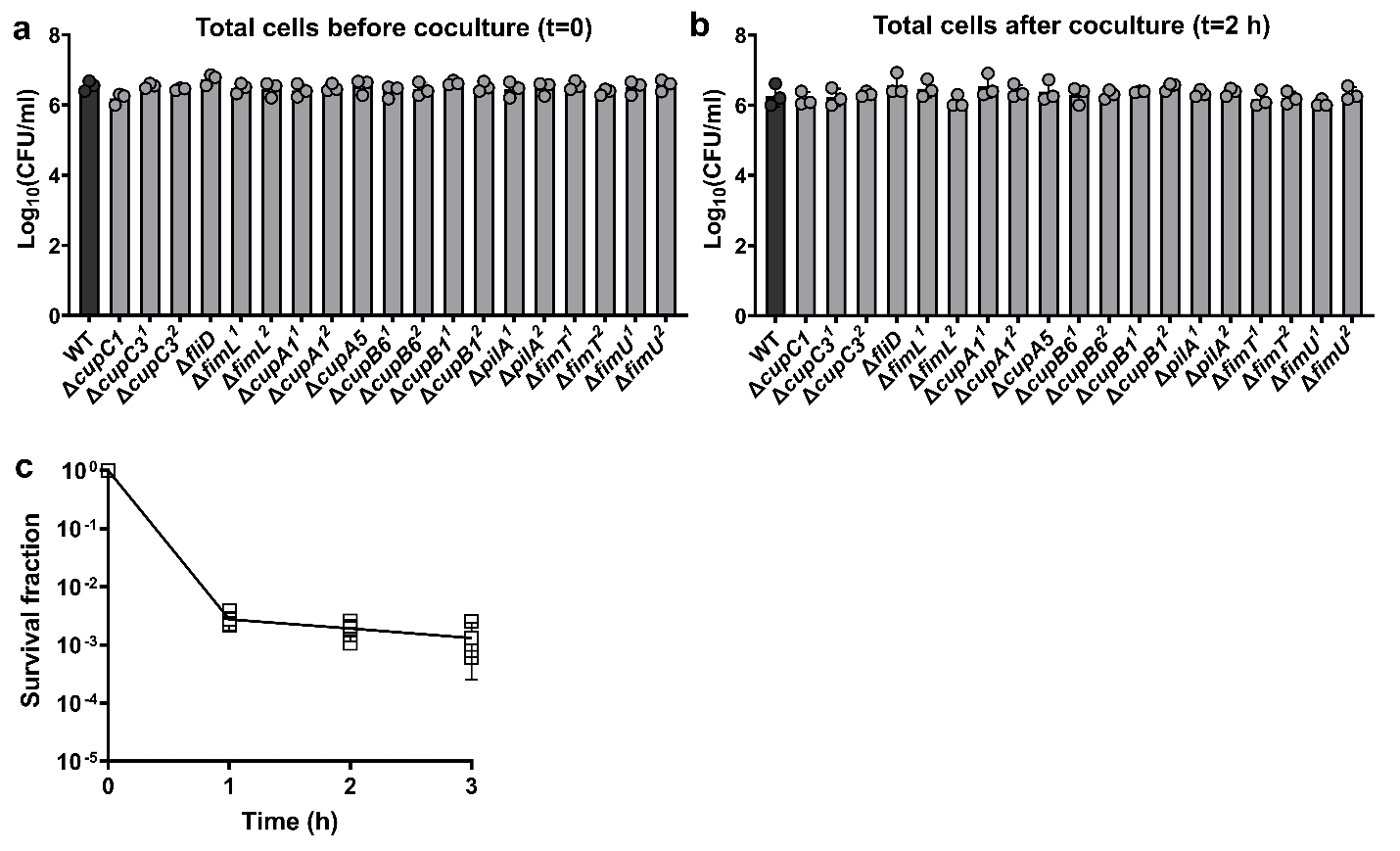


**Fig. S5: Clonogenic survival assays.** (**a-b**) The colony forming unit (CFU) counts of wild-type *P. aeruginosa* and the mutant strains. Bacterial cells were cocultured with lung cells (H1975) for 2 h at 37 °C. In order to quantify the number of colony-forming units for cells pre- (t=0) and post-interaction (t=2 h) between H1975 and *P. aeruginosa*, both swimming and attached bacterial cells were collected and plated onto Mueller-Hinton agar plates. (**c**) Time dependent bimodal killing curve. Wild-type *P. aeruginosa* cells were exposed to ofloxacin (10x MIC) for 3 hours in the presence of lung cells. Clonogenic survival assays were conducted at designated time points to determine the cell survival fractions. One-way ANOVA with Dunnett's post-test was utilized for the statistical analysis. The data for each time point is represented as the mean value ± standard deviation. N=3.


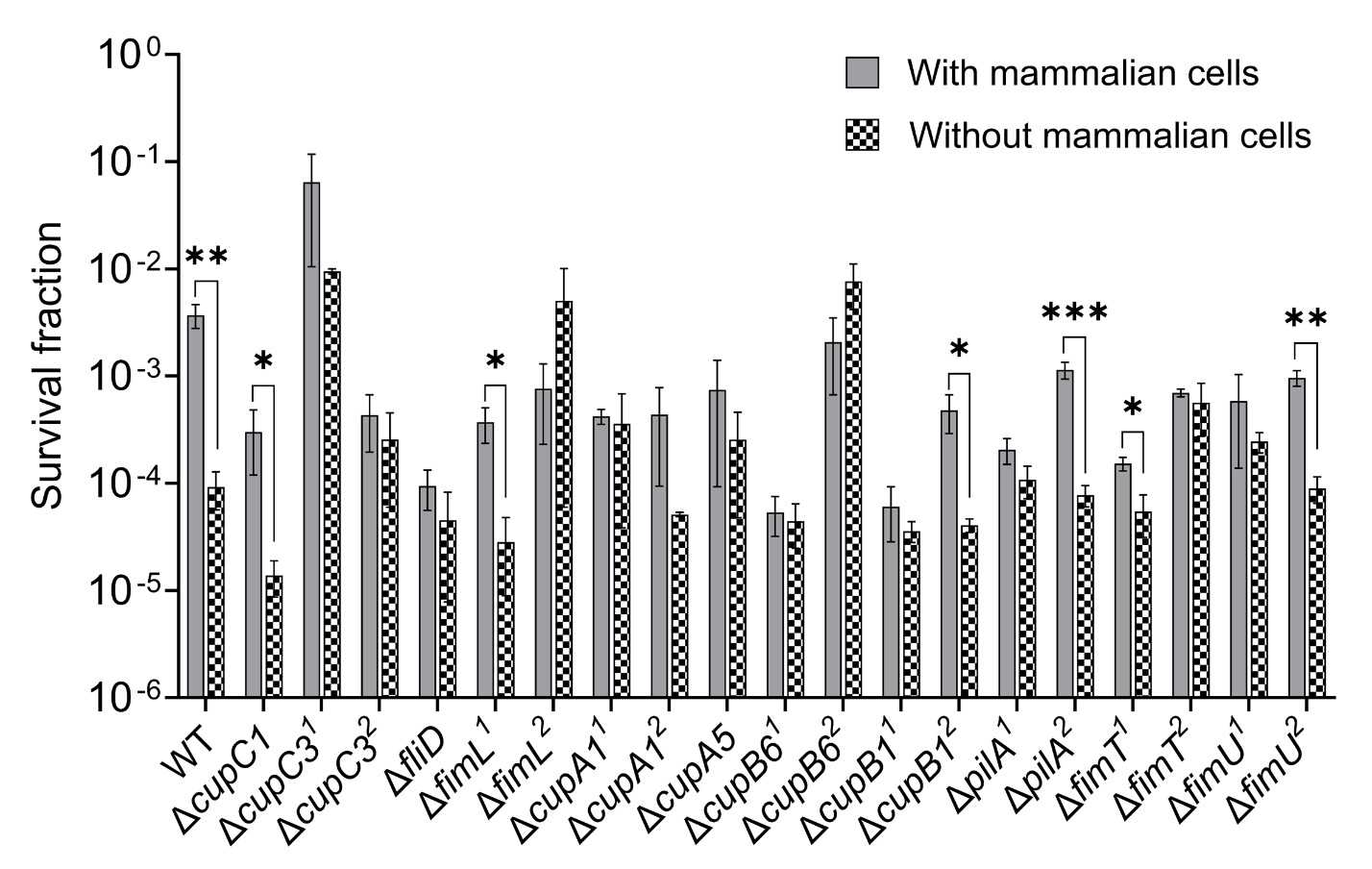


**Fig. S6: Clonogenic survival assays.** The colony forming unit (CFU) counts of wild-type *P. aeruginosa* and the mutant strains. Bacterial cells were cultured in the presence and absence of the lung cells (H1975) for 2 h at 37 °C. Then wild-type *P. aeruginosa* cells were exposed to ofloxacin (10x MIC) for 3 hours. Clonogenic survival assays were conducted at designated time points (before the treatment, t=0 and after the treatment, t=3 h) to determine the cell survival fractions. A two-tailed student t-test with unequal variance was used to determine statistical significance where the threshold P-value is 0.05. The data for each time point is represented as the mean value ± standard deviation. N=3.

**Supplementary Tables**

**Table S1: Bacterial strains, plasmids, mammalian cell lines, and oligonucleotides used in this study.**

| **A. Bacterial strains** | | | | **Source or reference** | |
| --- | --- | --- | --- | --- | --- |
| *Escherichia coli* K-12 MG1655 (wild type) | | | | In house | |
| *Pseudomonas aeruginosa* PAO1 (wild type) | | | | Manoil Lab | |
| *Pseudomonas aeruginosa* PAO1 Δ*cupC1* | | | | Manoil Lab | |
| *Pseudomonas aeruginosa* PAO1 Δ*cupC3^1^* | | | | Manoil Lab | |
| *Pseudomonas aeruginosa* PAO1 Δ*cupC3^2^* | | | | Manoil Lab | |
| *Pseudomonas aeruginosa* PAO1 Δ*fliD* | | | | Manoil Lab | |
| *Pseudomonas aeruginosa* PAO1 Δ*fimL^1^* | | | | Manoil Lab | |
| *Pseudomonas aeruginosa* PAO1 Δ*fimL^2^* | | | | Manoil Lab | |
| *Pseudomonas aeruginosa* PAO1 Δ*cupA1^1^* | | | | Manoil Lab | |
| *Pseudomonas aeruginosa* PAO1 Δ*cupA1^2^* | | | | Manoil Lab | |
| *Pseudomonas aeruginosa* PAO1 Δ*cupA5* | | | | Manoil Lab | |
| *Pseudomonas aeruginosa* PAO1 Δ*cupB6^1^* | | | | Manoil Lab | |
| *Pseudomonas aeruginosa* PAO1 Δ*cupB6^2^* | | | | Manoil Lab | |
| *Pseudomonas aeruginosa* PAO1 Δ*cupB1^1^* | | | | Manoil Lab | |
| *Pseudomonas aeruginosa* PAO1 Δ*cupB1^2^* | | | | Manoil Lab | |
| *Pseudomonas aeruginosa* PAO1 Δ*pilA^1^* | | | | Manoil Lab | |
| *Pseudomonas aeruginosa* PAO1 Δ*pilA^2^* | | | | Manoil Lab | |
| *Pseudomonas aeruginosa* PAO1 Δ*fimT^1^* | | | | Manoil Lab | |
| *Pseudomonas aeruginosa* PAO1 Δ*fimT^2^* | | | | Manoil Lab | |
| *Pseudomonas aeruginosa* PAO1 Δ*fimU^1^* | | | | Manoil Lab | |
| *Pseudomonas aeruginosa* PAO1 Δ*fimU^2^* | | | | Manoil Lab | |
| **B. Plasmids** | | | | **Source or reference** | |
| pMMB67EH-*yfp* | | | | Addgene (Catalog# 90104) | |
| pMMB67EH-*mCherry* | | | | This study | |
| **C. Mammalian cell lines** | | | | **Source or reference** | |
| Lung cancer cell (H1975) | | | | ATCC | |
| Melanoma cancer cell (A375) | | | | ATCC | |
| **C. Plasmid construction** | | | | | |
| pMMB67EH-*mCherry* | | The pMMB67EH-*yfp* plasmid was purchased from Addgene. The *mCherry* gene was amplified using forward and reverse primers with EcoRI and HindIII restriction enzyme cut sites, respectively. The pMMB67EH-*yfp* plasmid was double digested with HindIII and MluI to remove the *yfp* gene. Then, the digested *mCherry* gene and plasmid were ligated to generate pMMB67EH-*mCherry*. | | | |
| **D. Oligonucleotides to check mutant strains and plasmids construction** | | | | | |
| **Plasmid/deletion** | **Forward primer (5’ to 3’)** | | **Reverse primer (5’ to 3’)** | | **Source** |
| *mCherry*  (pMMB67EH-*mCherry*) | GCGCATGAATTCACT  TTAAGAAGGAGATA  TCATATGGTGAGCAA  GGGCGAGGAGGATA | | GCGCCTAAGCTTAGT  CTACTTGTACAGCTC  GTCCATGCCG | | Integrated DNA Technologies, Inc. |
| Δ*cupC1* | AAGCTGCTCAGGTTT  CTCCA | | ATAGATGCCAATACCG  ACGC | | Integrated DNA Technologies, Inc. |
| Δ*cupC3^1^* | TTTCCAACTACCAG  GCCAAC | | TCTTCTGCCGACTGGA  ACTT | | Integrated DNA Technologies, Inc. |
| Δ*cupC3^2^* | TTGTATACCTCCGC  TCAGGG | | TTGGCTTCATCCAGGT  ATCC | | Integrated DNA Technologies, Inc. |
| Δ*fliD* | CCAGTTCAAGAGTG  CGATCA | | TCGAGCTTCTTCTCGT  CGAT | | Integrated DNA Technologies, Inc. |
| Δ*fimL^1^* | TGCCGTTGTATATT  CTTGCG | | GCCACCAGGTAAAGC  AGTTC | | Integrated DNA Technologies, Inc. |
| Δ*fimL^2^* | GATCCAGGGCTACT  GCCTG | | GATAGAGGTTCTGTT  CGCGG | | Integrated DNA Technologies, Inc. |
| Δ*cupA1^1^* | CTCGTTCGAGTCTT  TACCGC | | AGAACAGCTTCAGGC  GATTG | | Integrated DNA Technologies, Inc. |
| Δ*cupA1^2^* | TGGACGATTATTGC  CTCACA | | GGTAGAACAGCTTCA  GGCGA | | Integrated DNA Technologies, Inc. |
| Δ*cupA5* | AGGTGGTTATCCGT  GTCCAG | | AGGATCAGTTCCTGG  GTCG | | Integrated DNA Technologies, Inc. |
| Δ*cupB6^1^* | GCTCTTCAAGCTGA  GGATCG | | CTGAACTGCTCGTTC  GACAA | | Integrated DNA Technologies, Inc. |
| Δ*cupB6^2^* | AAACGTATCCGCTG  CTGGT | | CTGAACTGCTCGTTC  GACAA | | Integrated DNA Technologies, Inc. |
| Δ*cupB1^1^* | CACTCTTCAACTGG  CTGGGT | | GTCGATGGGATCGTC  AACTT | | Integrated DNA Technologies, Inc. |
| Δ*cupB1^2^* | GAAACGTAGTAAGG  CGTCGG | | GGAACCAACTCCACT  TTCCA | | Integrated DNA Technologies, Inc. |
| Δ*pilA^1^* | ACTACATCTCCATCG  GCACC | | ACCCAGTTTCCTTGA  TCGTG | | Integrated DNA Technologies, Inc. |
| Δ*pilA^2^* | GGAATCAACGAGG  GCACC | | ACCCAGTTTCCTTGA  TCGTG | | Integrated DNA Technologies, Inc. |
| Δ*fimT^1^* | CTCGATCGGCACT  TCCTTC | | AGGAGCCTTTCCTTC  TTTGC | | Integrated DNA Technologies, Inc. |
| Δ*fimT^2^* | CGCCCACTACCAC  AACTCTT | | TGTCAGCTGCTTGAA  GTTCG | | Integrated DNA Technologies, Inc. |
| Δ*fimU^1^* | GAGAGGGCTGGCT  GCTTG | | GCGGTGTACTGGATG  GTCTT | | Integrated DNA Technologies, Inc. |
| Δ*fimU^2^* | CTGGCAACTGATCC  TCAACC | | GCGGTGTACTGGATG  GTCTT | | Integrated DNA Technologies, Inc. |

**Table S2: Comparing the distributions of average displacements between PA01 and mutant strains.**

| **Strains** | **Kolmogorov-Smirnov test, P value** |
| --- | --- |
| Δ*cupC1* | <0.000001 |
| Δ*cupC3^1^* | 0.174433 |
| Δ*cupC3^2^* | <0.000001 |
| Δ*fliD* | <0.000001 |
| Δ*fimL^1^* | <0.000001 |
| Δ*fimL^2^* | <0.000001 |
| Δ*cupA1^1^* | <0.000001 |
| Δ*cupA1^2^* | <0.000001 |
| Δ*cupA5* | <0.000001 |
| Δ*cupB6^1^* | <0.000001 |
| Δ*cupB6^2^* | <0.000001 |
| Δ*cupB1^1^* | <0.000001 |
| Δ*cupB1^2^* | <0.000001 |
| Δ*pilA^1^* | 0.008441 |
| Δ*pilA^2^* | <0.000001 |
| Δ*fimT^1^* | <0.000001 |
| Δ*fimT^2^* | <0.000001 |
| Δ*fimU^1^* | <0.000001 |
| Δ*fimU^2^* | <0.000001 |
| *E. coli* MG1655 | <0.000001 |

**Table S3: Percentage of attached and unattached/motile cells in P. aeruginosa strains.**

| **Strain** | **% attached** | **% unattached/motile** |
| --- | --- | --- |
| *P. aeruginosa PA01* | 2.54 | 97.46 |
| *P. aeruginosa ΔcupC1* | 6.10 | 93.90 |
| *P. aeruginosa ΔcupC3^1^* | 2.80 | 97.20 |
| *P. aeruginosa ΔcupC3^2^* | 3.62 | 96.38 |
| *P. aeruginosa ΔfliD* | 0.86 | 99.14 |
| *P. aeruginosa ΔfimL^1^* | 2.51 | 97.49 |
| *P. aeruginosa ΔfimL^2^* | 4.64 | 95.36 |
| *P. aeruginosa ΔcupA1^1^* | 3.76 | 96.24 |
| *P. aeruginosa ΔcupA1^2^* | 2.64 | 97.36 |
| *P. aeruginosa ΔcupA5* | 3.45 | 96.55 |
| *P. aeruginosa ΔcupB6^1^* | 3.25 | 96.75 |
| *P. aeruginosa ΔcupB6^2^* | 5.69 | 94.31 |
| *P. aeruginosa ΔcupB1^1^* | 4.55 | 95.45 |
| *P. aeruginosa ΔcupB1^2^* | 3.47 | 96.53 |
| *P. aeruginosa ΔpilA^1^* | 2.54 | 97.46 |
| *P. aeruginosa ΔpilA^2^* | 2.27 | 97.73 |
| *P. aeruginosa ΔfimT^1^* | 2.12 | 97.88 |
| *P. aeruginosa ΔfimT^2^* | 3.89 | 96.11 |
| *P. aeruginosa ΔfimU^1^* | 2.85 | 97.15 |
| *P. aeruginosa ΔfimU^2^* | 3.28 | 96.72 |

**Table S4: MIC levels of ofloxacin for the strains used in this study.**

| **Bacterial strain** | **Bactericidal antibiotics** | **MIC Range (µg/ml)** | **Clonogenic survival assay concentration (µg/ml)** |
| --- | --- | --- | --- |
| *Pseudomonas aeruginosa* PAO1 (wild type) | Ofloxacin | 0.75-1.0 | 10 |
| *Pseudomonas aeruginosa* PAO1 Δ*cupC1* | Ofloxacin | 0.75-1.0 | 10 |
| *Pseudomonas aeruginosa* PAO1 Δ*cupC3^1^* | Ofloxacin | 1.5-2.0 | 10 |
| *Pseudomonas aeruginosa* PAO1 Δ*cupC3^2^* | Ofloxacin | 0.75-1.0 | 10 |
| *Pseudomonas aeruginosa* PAO1 Δ*fliD* | Ofloxacin | 0.75-1.0 | 10 |
| *Pseudomonas aeruginosa* PAO1 Δ*fimL^1^* | Ofloxacin | 0.75-1.0 | 10 |
| *Pseudomonas aeruginosa* PAO1 Δ*fimL^2^* | Ofloxacin | 0.75-1.0 | 10 |
| *Pseudomonas aeruginosa* PAO1 Δ*cupA1^1^* | Ofloxacin | 0.75-1.0 | 10 |
| *Pseudomonas aeruginosa* PAO1 Δ*cupA1^2^* | Ofloxacin | 0.75-1.0 | 10 |
| *Pseudomonas aeruginosa* PAO1 Δ*cupA5* | Ofloxacin | 0.75-1.0 | 10 |
| *Pseudomonas aeruginosa* PAO1 Δ*cupB6^1^* | Ofloxacin | 0.75-1.0 | 10 |
| *Pseudomonas aeruginosa* PAO1 Δ*cupB6^2^* | Ofloxacin | 0.75-1.0 | 10 |
| *Pseudomonas aeruginosa* PAO1 Δ*cupB1^1^* | Ofloxacin | 0.75-1.0 | 10 |
| *Pseudomonas aeruginosa* PAO1 Δ*cupB1^2^* | Ofloxacin | 0.75-1.0 | 10 |
| *Pseudomonas aeruginosa* PAO1 Δ*pilA^1^* | Ofloxacin | 0.75-1.0 | 10 |
| *Pseudomonas aeruginosa* PAO1 Δ*pilA^2^* | Ofloxacin | 0.75-1.0 | 10 |
| *Pseudomonas aeruginosa* PAO1 Δ*fimT^1^* | Ofloxacin | 0.75-1.0 | 10 |
| *Pseudomonas aeruginosa* PAO1 Δ*fimT^2^* | Ofloxacin | 0.75-1.0 | 10 |
| *Pseudomonas aeruginosa* PAO1 Δ*fimU^1^* | Ofloxacin | 0.75-1.0 | 10 |
| *Pseudomonas aeruginosa* PAO1 Δ*fimU^2^* | Ofloxacin | 0.75-1.0 | 10 |

**Table S5: Chemicals used in this study.**

| **Chemicals** | **Purity (%)** | **Source** | **Catalog number** |
| --- | --- | --- | --- |
| Isopropyl ß-D-1 thiogalactopyranoside | >99 | Fisher Scientific | BP1755-10 |
| Gentamicin sulfate | **>**99 | VWR | 0304-100G |
| Ofloxacin | 98 | Fisher Scientific | AC455670050 |
| HEPES hemisodium salt | 98 | ThermoFisher Scientific | J61830.36 |
