# Supplementary material for: Uncovering bacterial-mammalian cell interactions via single-cell tracking
Source: BMC Biol. 2024 Nov 11;22:256. doi: 10.1186/s12915-024-02056-z (PMC11552363; doi:10.1186/s12915-024-02056-z)
Supplement: Supplementary file 2 — Additional file 2. Movie S1. Bacterial cell trajectories. Trajectories of P. aeruginosa PA01 near the lung cell for (a) an attached bacterium, (b) a surface-bound bacterium rotating in the clockwise direction, (c) a bacterium moving on the mammalian cell surface, and (d) a bacterium approaching the H1975 cell and abruptly turning away. Fluorescent images depicting bacterial cells were exclusively featured in the movies to ensure enhanced resolution. The mammalian cells in the movies were depicted in Supplementary Figure S1a-d, delineated by blue circles for clarity. Movie S2. Representative movies for bacterial strains. (a-b) Untracked and tracked Escherichia coli MG1655 cells, respectively. Single cell tracking in panel “b”, highlighted in red circles, is performed using a MATLAB code. The tracked video (panel b) is ~8 times slower than its original version (panel a). (c-d) Untracked and tracked P. aeruginosa wild-type (WT) cells, respectively. Single cell tracking in panel “d”, highlighted in red circles, is performed using a MATLAB code. The tracked video (d) is ~8 times slower than its original version (panel c). Movie S3. Representative movie of P. aeruginosa ΔfliD strain. (a-b) Untracked and tracked P. aeruginosa ΔfliD strain, respectively. Single cell tracking in panel “b”, highlighted in red circles, is performed using a MATLAB code. The tracked video is ~8 times slower than its original version (panel a). [file 12915_2024_2056_MOESM2_ESM.zip › Movie S2cd.pptx]

## Slide 1
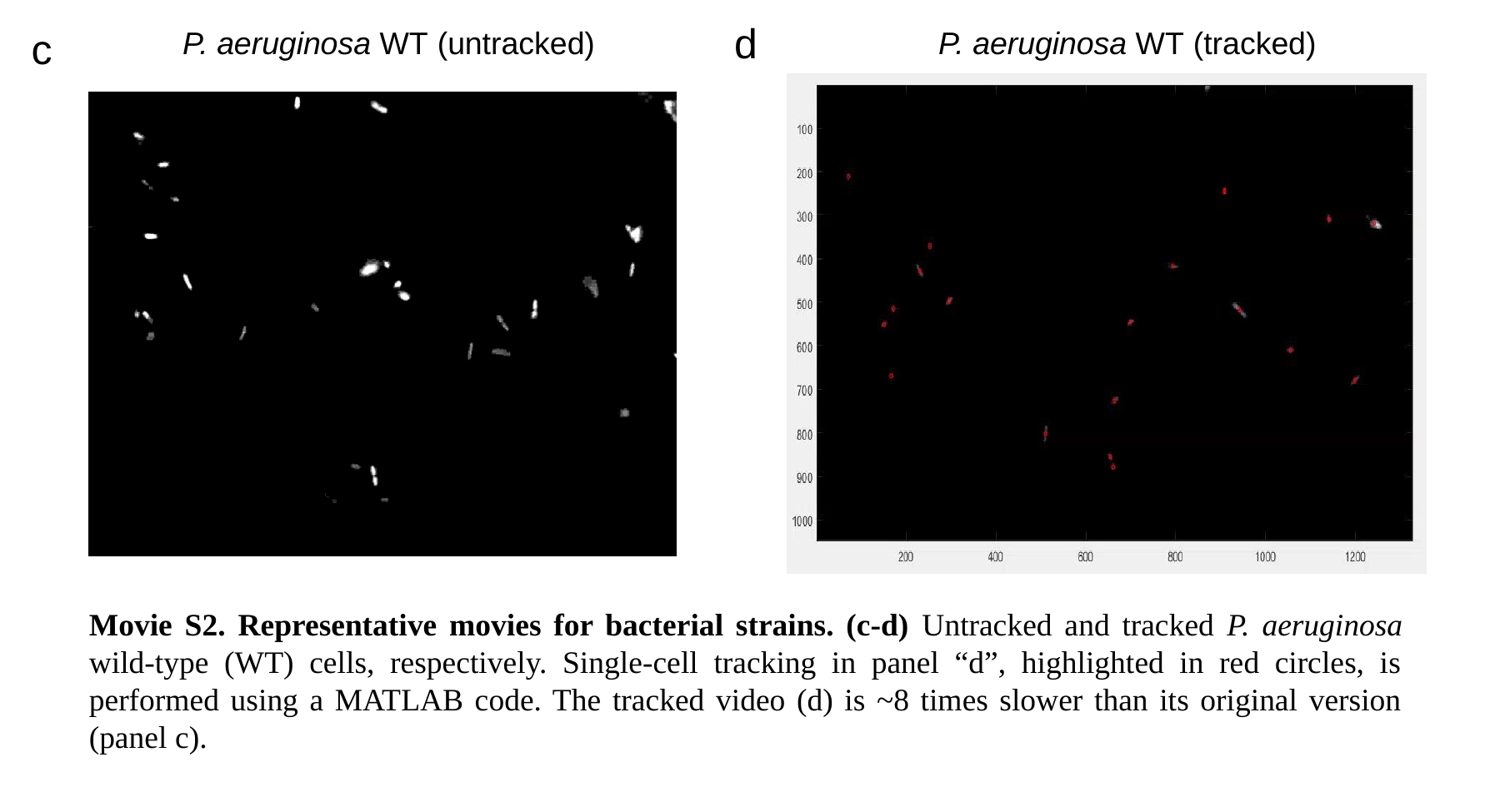

d
P. aeruginosa WT (untracked)
P. aeruginosa WT (tracked)
c
Movie S2. Representative movies for bacterial strains. (c-d) Untracked and tracked P. aeruginosa wild-type (WT) cells, respectively. Single-cell tracking in panel “d”, highlighted in red circles, is performed using a MATLAB code. The tracked video (d) is ~8 times slower than its original version (panel c).
